# Design and analysis of CRISPR‐based underdominance toxin‐antidote gene drives

**DOI:** 10.1111/eva.13180

**Published:** 2020-12-21

**Authors:** Jackson Champer, Samuel E. Champer, Isabel K. Kim, Andrew G. Clark, Philipp W. Messer

**Affiliations:** ^1^ Department of Computational Biology Cornell University Ithaca New York USA; ^2^ Department of Molecular Biology and Genetics Cornell University Ithaca New York USA

**Keywords:** confinement, CRISPR, gene drive, genetic engineering, modeling, toxin‐antidote, underdominance

## Abstract

CRISPR gene drive systems offer a mechanism for transmitting a desirable transgene throughout a population for purposes ranging from vector‐borne disease control to invasive species suppression. In this simulation study, we assess the performance of several CRISPR‐based underdominance gene drive constructs employing toxin‐antidote (TA) principles. These drives disrupt the wild‐type version of an essential gene using a CRISPR nuclease (the toxin) while simultaneously carrying a recoded version of the gene (the antidote). Drives of this nature allow for releases that could be potentially confined to a desired geographic location. This is because such drives have a nonzero‐invasion threshold frequency required for the drive to spread through the population. We model drives which target essential genes that are either haplosufficient or haplolethal, using nuclease promoters with expression restricted to the germline, promoters that additionally result in cleavage activity in the early embryo from maternal deposition, and promoters that have ubiquitous somatic expression. We also study several possible drive architectures, considering both “same‐site” and “distant‐site” systems, as well as several reciprocally targeting drives. Together, these drive variants provide a wide range of invasion threshold frequencies and options for both population modification and suppression. Our results suggest that CRISPR TA underdominance drive systems could allow for the design of flexible and potentially confinable gene drive strategies.

## 1 INTRODUCTION

### 1.1 Gene drive systems

It is possible to engineer alleles that can spread at a higher rate than expected under Mendelian inheritance by biasing their own rate of transmission. These are known as “gene drives” (Alphey, 2014; Burt, 2014; Champer et al., 2016; Deredec et al., 2011; Esvelt et al., 2014; Noble et al., 2017; Unckless et al., 2015). While there are many examples for naturally occurring alleles with super‐Mendelian transmission, purpose‐engineered gene drive systems are gaining increasing interest as methods to spread desirable transgenic packages throughout a species of interest. The potential applications of such drives fall broadly into two categories: those designed for population modification and those designed for population suppression (Alphey, 2014; Burt, 2014; Champer et al., 2016; Esvelt et al., 2014). A population modification system may, for example, be designed to alter a trait in mosquitoes that results in reduced transmission of certain diseases. Another example of a modification system would be one designed to help rescue an endangered species from a disease that is spreading too rapidly for natural evolutionary processes to counter (Burt, 2014; Champer et al., 2016; Esvelt et al., 2014). Suppression gene drives, on the other hand, could be intended to result in the eradication of an invasive species, an agricultural pest, or a disease vector (Alphey, 2014; Burt, 2014; Champer et al., 2016; Esvelt et al., 2014).

CRISPR homing drives have recently been the subject of intensive research efforts. These drives utilize CRISPR‐Cas9 to cleave the wild‐type allele in heterozygotes at a guide RNA (gRNA) target site. The severed DNA end is then repaired by homology‐directed repair during which the cell uses the drive‐carrying chromosome as a template, resulting in a cell homozygous for the drive allele. This process is intended to occur in germline cells, so that drive carriers will pass the drive on to their offspring at an increased rate. Homing drives have already been developed in a variety of species (Adolfi et al., 2020; Champer et al., 2017; Gantz et al., 2015; Galizi et al., 2016; Grunwald et al., 2019; Yan & Finnigan, 2019). While resistance alleles have proven a substantial obstacle for these drives, two studies have recently demonstrated a successful modification drive in flies (Champer, Yang, et al., 2020) and a successful suppression drive in mosquitoes (Kyrou et al., 2018).

One problematic feature of homing‐type systems is that they are so‐called “global” drives. Unless they have a very high fitness cost, the introduction of even a small number of drive‐carrying individuals will likely result in the drive spreading throughout an entire population (Noble et al., 2018). For target species such as mosquitoes that are capable of long‐distance migration by piggybacking on human‐based modes of travel (Eritja et al., 2017) as well other means such as high‐altitude air currents (Huestis et al., 2019), this implies that a single release of a homing drive could potentially result in the drive spreading throughout the entire range of the species. In some scenarios, this may be desirable, such as when the drive in question is engineered for disease prevention and when there exists a high degree of social approval. However, this perhaps limits this strategy to a few especially harmful species, such as disease‐carrying ticks and mosquitoes. For many other potential applications, confinement to a target area would be highly desirable.

### 1.2 Underdominance gene drives

The principle of underdominance is that drive/wild‐type heterozygotes are less successful than either drive or wild‐type homozygotes. This is generally expected to result in a nonzero‐invasion threshold frequency for an underdominance‐based gene drive even when it does not carry any additional fitness costs (note that whenever we refer to the fitness costs of a drive, we mean additional costs that do not result from the drive mechanism itself, i.e., the disruption of wild‐type alleles leading to nonviability). By contrast, most other types of gene drives (even frequency‐dependent ones) have a zero‐invasion threshold, sometimes even if the drive carries a fitness cost (depending on the specific type of drive). An “invasion threshold” represents the introduction frequency of drive‐carrying individuals above which the drive is expected to increase in frequency either to fixation or to some equilibrium frequency (Altrock et al., 2010, 2011; Champer, Zhao, et al., 2020; Dhole et al., 2018; Edgington & Alphey, 2018; Marshall & Hay, 2012a). When introduced below this frequency, the drive is expected to be lost from the population. Drives with nonzero‐invasion thresholds even in the absence of fitness costs are often termed “local” drives, though the exact degree of localization that can be expected depends on the threshold and numerous other ecological factors. In a scenario where two demes are linked by migration, and assuming panmixia within each deme, local drives can be successfully confined to one of the demes if the migration rate between them is below a critical threshold (Altrock et al., 2010, 2011; Champer, Zhao, et al., 2020; Dhole et al., 2018; Edgington & Alphey, 2018; Marshall & Hay, 2012a). This “migration threshold”, is correlated with the invasion threshold frequency, but also accounts for the fact that even a low rate of migration could eventually lead to the drive exceeding its invasion threshold in the second deme when drive alleles can accumulate over time (Altrock et al., 2010, 2011; Champer, Zhao, et al., 2020; Dhole et al., 2018; Edgington & Alphey, 2018; Marshall & Hay, 2012a).

Several designs have already been proposed for gene drives that could potentially be confined. Examples of such drives include the *Medea* toxin‐antidote (TA) system (Chen et al., 2007), variations thereof (Akbari et al., 2013), *Wolbachia* TA elements (Shropshire & Bordenstein, 2019), reciprocal chromosomal translocations (Foster et al., 1972), and a single‐locus underdominance TA system called RPM‐Drive (Reed et al., 2018; Reeves et al., 2014). These (Altrock et al., 2010, 2011; Barton, 1979; Barton & Hewitt, 1989; Champer, Zhao, et al., 2020; Davis et al., 2001; Edgington & Alphey, 2017, 2018; Huang et al., 2009, 2011; Khamis et al., 2018; Láruson & Reed, 2016; Magori & Gould, 2006) and other (Gould et al., 2008; Marshall, 2011; Marshall & Hay, 2011, 2012a, 2012b, 2014; Marshall et al., 2011) TA designs have been modeled computationally. Yet both demonstrated and proposed systems have proven difficult to successfully engineer in species of interest due to the need for highly specific targets, promoters, and RNAi elements, methods that tend to introduce high fitness costs, the need to fine‐tune gene repression, or complicated engineering methods. All these proposed drives with invasion thresholds thus far have focused on population modification strategies, while there has not yet been a proposed design for a threshold‐dependent suppression drive.

### 1.3 CRISPR toxin‐antidote gene drives

CRISPR TA gene drives have recently been devised as an alternative class of drive systems that are substantially less vulnerable to resistance than CRISPR homing drives (largely because multiplexing can prevent the formation of function‐preserving resistance alleles (Champer, Kim et al., 2020) and because such drives would not suffer from loss of efficiency as would homing‐type drives with multiple gRNAs (Champer, Oh, et al., 2020) and often promise easier construction as compared to RNAi‐based systems (Champer, Kim, et al., 2020; Champer, Lee, et al., 2020; Oberhofer et al., 2019). Several of the suggested designs also have invasion thresholds, meaning that they are unlikely to spread through a population unless the drive allele is present above a critical frequency. In a CRISPR‐based TA system, the “toxin” is a Cas9 element with gRNAs programmed to cut an essential gene on the wild‐type chromosome, where cleavage‐repair will typically result in a disrupted version of the target gene (Figure S1). The “antidote” element is a functioning copy of the target gene that is located within the drive allele and is recoded to no longer match the drive's gRNAs so that it is not subject to disruption by the drive. Thus, individuals who inherit only a toxin‐disrupted allele suffer from a toxic effect, while individuals who only inherit the drive, or who inherit both a disrupted allele as well as the antidote, do not experience the deleterious toxic effect. By this mechanism, the relative frequency of the drive should increase over time as wild‐type alleles are removed from the population (Champer, Kim, et al., 2020).

Two such CRISPR‐based TA systems, termed TARE (Toxin‐Antidote Recessive Embryo drive) and Cleave and Rescue (ClvR), have already been demonstrated in *Drosophila*—both were able to rapidly spread through cage populations (Champer, Lee, et al., 2020; Oberhofer et al., 2019). In a recent study, we have modeled several additional designs by varying the nature of the target gene and the expression profile of the drive promoter, showing that these designs can in principle be used for both population modification and suppression strategies (Champer, Kim, et al., 2020). However, most of these designs were so‐called “regional” drives, which have a nonzero introduction threshold only when the drive allele carries fitness costs in addition to those imposed by the drive mechanism itself (i.e., costs associated with the disruption of wild‐type alleles). While it seems a reasonable assumption that any gene drive system should have at least some such additional fitness cost (due to expression of the large Cas9 protein, for example), the thresholds of these systems may still be too low for applications where more stringent confinement is desired.

In this study, we propose and model several new designs for CRISPR‐based TA systems that can be configured to function as “local” drives by employing underdominance principles. This is in contrast to previously considered “regional” CRISPR TA systems that do not employ underdominance mechanisms and which generally have a nonzero‐invasion threshold frequency only if the drive has additional fitness costs (Champer, Kim, et al., 2020

; Champer, Lee, et al., 2020; Oberhofer et al., 2019), or “global” TA systems that have a zero‐invasion threshold even under a wide range of fitness costs (Champer, Kim, et al., 2020). These local drives are characterized by nonzero‐invasion threshold frequencies even without additional fitness costs, thereby offering a higher degree of confinement than regional drives. We first discuss the general components of these drives, including the nuclease promoter, the type of target gene and rescue element, and the different architectures in which these elements can be arranged. This is followed by a consideration of specific drive systems and computational analysis of their expected performance through simulations in a simple panmictic population model. We focus on a selection of several such systems and variants with particularly unique or interesting properties and which seem plausible to construct with current technology. These drives feature a wide span of invasion thresholds and include both population modification and suppression drives.

## 2 METHODS

### 2.1 Population model

Our simulation model considers a single panmictic population of sexually reproducing diploids with nonoverlapping generations. Each individual is specified by its genotype at the drive locus (or drive loci for strategies involving more than one locus) and any additional potential drive target loci, if different from the drive loci.

The fitness of an individual is influenced by its genotype, as defined for the specific drive strategy. In this manuscript, we refer to the *fitness* (*ω*) of a drive individual as the relative (to wild‐type) fitness due to costs directly imposed by drive alleles (e.g., due to expression of the Cas9 protein, the presence of certain other drive elements, or a potential payload). As an approximation, we assume that reductions in fitness due to the presence of drive alleles only affect female fecundity and male mating success, though egg‐to‐adult viability would also likely be affected (modeling drive allele impact using various combinations of fecundity cost, mating success reduction, and viability reduction results in similar model outcomes). Note that in this definition, *fitness* does not take into account any offspring of an individual that are rendered nonviable by the drive mechanism itself (based on the disruption of wild‐type alleles), nor the possibility that drive individuals themselves may be sterile when carrying a suppression type drive. Wild‐type individuals are assumed to have a *fitness* of 1. We generally assume in our model that fitness costs are due to expression of drive components such as Cas9 or from effects of a payload gene. In that case, it is reasonable to assume that fitness costs from a drive are multiplicative: if drive homozygotes have a fitness *ω*, drive heterozygotes will have fitness $\sqrt{\omega }$. To make it easier to compare the drives, in drive systems that consist of multiple drive alleles at different loci, only alleles at one drive locus are allowed to carry additional fitness costs (in suppression drives, if one type of drive allele disrupts a fertility gene with its presence, this is the allele which is modeled as having a fitness cost).

In any given generation, the state of the population is defined by the numbers of male and female adults of each genotype. To determine the state of the population in the next generation, each female first selects a random candidate among all males in the population. The candidate is then accepted for mating at a rate equal to his fitness value (e.g., a male with fitness 0.5 would be selected half the time). If the candidate is rejected, the female chooses another random candidate; if the female rejects ten candidates, she does not reproduce (we selected a high number for this level so that most females will successfully find a mate). After a mate has been selected, we set the fecundity of the female (i.e., her expected number of offspring) to twice her fitness value, multiplied by a density‐dependent scaling factor σ=β/((β‐1)×N/K+1). Here, *β* is the maximum low‐density growth rate, *N* is the current population size, and *K* is the carrying capacity of the population. As default values for our model, we used *β* = 10 to provide a substantial increase to the growth rate at low density and *K* = 100,000. A *β* value of 10 means that the population is able to experience a 10‐fold growth rate per generation at very low density, representing reduced competition at lower population sizes. The actual number of offspring for the female is then determined by a draw from a binomial distribution with 50 trials and p=ω×σ/25, yielding a maximum of 50 offspring and an average of two offspring for a female with fitness ω = 1.0 in a population at carrying capacity (before accounting for potential sterility or nonviability of offspring due to the drive mechanism). These parameters are expected to result in logistic growth dynamics where the population equilibrates toward carrying capacity when disturbed, except in the presence of a sufficiently robust suppression drive system. The large initial population size usually minimizes stochastic effects in the simulations, though such effects tend to become more pronounced in simulations of suppression type drives as the population dwindles.

In simulations examining the migration thresholds in a 2‐deme model, the population was evenly divided into two demes with *K* = 50,000, with the migration frequency specifying the rate at which offspring generated in one deme are placed in the other deme.

Each offspring generated is assigned a random sex, and its genotype is determined by randomly selecting one allele from each parent, with the possibility that wild‐type alleles may be converted into disrupted alleles by drive activity. If a parent has a drive allele, it disrupts a wild‐type target allele from that parent at a rate corresponding to the germline cut rate (which is variable and based on the level of Cas9 expression from its promoter). Next, additional disruption can occur in the embryo, where each of the mother's drive alleles may disrupt wild‐type target alleles at a rate dictated by the type of promoter utilized by the drive. Wouldbe offspring with nonviable genotypes are then removed (see Supplemental tables for several examples).

At the outset of the simulations, a percentage of individuals in the simulation are set as carriers of the selected gene drive system. Alternatively, a fixed number of gene drive carriers are added to the population each generation rather than an initial one‐time release. Introduced drive‐carrying individuals are homozygous for modification drives or heterozygous for suppression drives. The simulation is then run for 100 generations, or 150 generations for 2‐deme simulations, with several metrics of drive performance tracked. All models were implemented in the SLiM simulation framework (Haller & Messer, 2019). See the supplemental information for a full list of parameters and variables.

### 2.2 Data output

At each generational step in our simulations, we record the frequency of the drive allele(s) in the population, the percentage of individuals that are drive carriers, the population size, and the genetic load of the drive on the population. The genetic load that the drive imposes on the population in a given generation is defined as 1‐*N*_act_/*N*_exp_, where *N*_act_ is the actual number of individuals observed in the next generation and *N*_exp_ is the number expected if all individuals were wild type (according to our logistic growth model). By taking the average of the genetic load over several generations after the drive has reached fixation or equilibrium, we can assess the reproductive burden that a drive imposes on a population.

We used a numerical approach to estimate the required introduction threshold of a drive. In particular, we considered a given introduction frequency to be above the threshold if the drive increased in frequency during any ten consecutive generations within the 50 generations following the introduction of the drive. The final thresholds assigned to the drives are the lowest introduction rates where, in half or more simulations, this condition was satisfied.

### 2.3 Data generation and software

Simulations were run on the computing cluster of the Department of Computational Biology at Cornell University. All simulations were run using SLiM version 3.3. Data processing, analysis, and figure preparation were performed in Python 3.7.4. The SLiM program and parameter files allowing the reader to reproduce all simulations presented here are available on GitHub (https://github.com/MesserLab/TA‐Underdominance‐Drives). Each point shown in the heatmap figures represents an averaged result from 20 simulations.

## 3 RESULTS

### 3.1 Basic components and specifications of the TA systems analyzed

#### 3.1.1 Drive payload and fitness costs

Drives can carry a payload gene or have components that produce fitness costs, which we modeled as multiplicative based on the number of drive alleles (at just one drive locus for multi‐locus systems for ease of comparison between drive types). Unless otherwise specified, idealized drives (line graph figures), representing perfectly efficient systems or systems with only a single imperfect parameter, are assumed to carry no fitness cost. Nonidealized drives (heatmap figures), representing potentially more realistic and achievable systems, are assumed to have a fitness value of *ω* = 0.95.

#### 3.1.2 Nuclease promoter

The choice of promoter regulates the expression of the Cas9 (or another nuclease) and thus determines the rate and timing at which the drive disrupts wild‐type target genes. The choice of promotor can thereby affect whether cutting occurs primarily in the germline or also during early embryo development due to maternally deposited Cas9. Unless otherwise specified, an idealized germline promoter (G promoter) would result in 100% cutting activity in the germline and no activity in the early embryo from maternally deposited Cas9, while an idealized germline and embryo promoter (GE promoter) would have 100% cutting activity at both stages. For nonidealized forms, we assume in our model that germline cut rate is 99% in both cases, as seems to be the case with most experimentally tested drive promoters (Champer et al., 2018; Hammond et al., 2018), while embryo activity is 5% for a germline‐restricted promoter (Hammond et al., 2018; Kyrou et al., 2018) and 95% for a promoter that also has high activity in the early embryo (Champer, Chung, et al., 2019; Hammond et al., 2015). In principle, both the germline and embryo cut rate could vary dynamically for promoters (with mosaic cutting an additional possibility in the early embryo). We limit our consideration of promoters to these default parameters for this study, except for TADE underdominance variants below (see a previous study (Marshall, 2011) on similar TA systems for the effects of variation in cut rates). In general, reduced cut rates will reduce the efficiency of the drive, slowing its spread and increasing the required introduction threshold (Marshall, 2011).

A promoter with somatic expression (Champer et al., 2018; Hammond et al., 2015; GES promoter) additionally induces Cas9 expression in nongermline cells, leading to the formation of disrupted wild‐type target alleles if a drive allele is present. We model such promoters as having activity in the germline and embryo as above, but individuals with drive alleles and wild‐type target sites are then considered to also have disrupted target sites when determining viability and fitness in our simulation model. This represents a GES promoter that induces substantial somatic Cas9 expression.

#### 3.1.3 Target gene and rescue element

Loss‐of‐function alleles of genes targeted by a TA system can be either dominant (haploinsufficient/haplolethal) or recessive (essential but haplosufficient). In TA systems with haplolethal targets (e.g., TADE, Toxin‐Antidote Dominant Embryo drive), an individual is nonviable if it has less than two functioning copies of the gene, unless drive rescue provides an equivalent level of gene expression. An example of a haplolethal gene target is *RpL35A* in *Drosophila* (Champer, Yang, et al., 2020). If the drive target is essential but haplosufficient (e.g., TARE, Toxin‐Antidote Recessive Embryo drive), the individual is fully viable if it has at least one functioning copy of the gene, or the equivalent provided by drive rescue. Haplosufficient genes are likely plentiful in most organisms, with one example being *hairy*, as used in a previously constructed TARE drive (Marshall, 2011). “Regional” TARE and TADE drives (including a suppression form of the latter) have been modeled previously with both G and GE promoters (Champer, Kim, et al., 2020; Champer, Lee, et al., 2020; Oberhofer et al., 2019), but in this study, we present additional unique configurations of drives with TARE and TADE elements.

The rescue provided by the drive could be equivalent to the functionality of the wild‐type allele, or, by using different regulatory sequences or otherwise modifying the rescue portion of the drive, could be configured to provide half the functionality of the wild‐type allele (e.g., TAHRE, Toxin‐Antidote Half rescue Recessive Embryo drive—two copies of the drive are equivalent to a single wild‐type copy of the target gene). Similarly, by using two rescue gene copies in a single drive allele, the drive could provide double functionality (e.g., TADDE, Toxin‐Antidote Double‐rescue Dominant Embryo drive—one drive allele is equivalent to two copies of the wild‐type target allele). TADDE drive has been modeled previously in a simple configuration that resulted in a “regional” drive (Champer, Kim, et al., 2020).

Four meaningful permutations of these systems can be envisioned: double rescue with a haplolethal target (TADDE), single rescue with a haplolethal target (TADE), single rescue with a haplosufficient target (TARE), and half rescue with a haplosufficient target (TAHRE). Generally, TARE and TADDE systems are suitable for population modification but lack the ability to impose a substantial genetic load on a population for suppression (Champer, Kim, et al., 2020). TADE (Champer, Kim, et al., 2020) and TAHRE systems can optionally introduce strong genetic loads for population suppression (as we will show below), though suppression systems tend to act more slowly than modification systems.

#### 3.1.4 Drive architecture

A drive allele can be a “same‐site” allele where the drive is co‐located with the gene that it provides rescue to, or it can be “distant‐site” where the drive provides rescue for a distant gene. Some types of distant‐site drives can strongly suppress a population if the drive is located in an essential but haplosufficient female fertility gene, disrupting the gene with its presence. A drive homozygous female would therefore be sterile. An example of such a suitable location is an exon of *doublesex* in *Anopheles* (Champer, Yang, et al., 2020; in this case, the drive both targets and is co‐located at the *doublesex* exon). Both same‐site and distant‐site alleles can also be used for population suppression if additional gRNAs that target the female fertility gene are included in the drive (without a recoded rescue element), even if the drive itself is not located in the fertility gene. Note that in either of these cases, the gene (which the drive disrupts with its presence or with additional gRNAs targeting the gene) can also be male‐specific, or affect viability instead of fertility, while still allowing the drive to strongly suppress populations.

Drive arrangements can further be classified into three types: single‐locus systems with a single drive allele [all previously considered CRISPR TA systems used such a configuration (Champer, Kim, et al., 2020; Champer, Lee, et al., 2020; Oberhofer et al., 2019)], single‐locus systems with two types of drive alleles, and multi‐locus (usually two) systems with a different type of drive allele at each site. In the single‐locus system with a single drive allele, the drive targets and provides rescue for the same gene. In the other two systems, the first drive allele targets the gene that the second drive allele provides rescue for, and vice versa.

### 3.2 Design and analysis of underdominance TA systems

#### 3.2.1 1‐locus 2‐drive TARE

**Figure 1:**
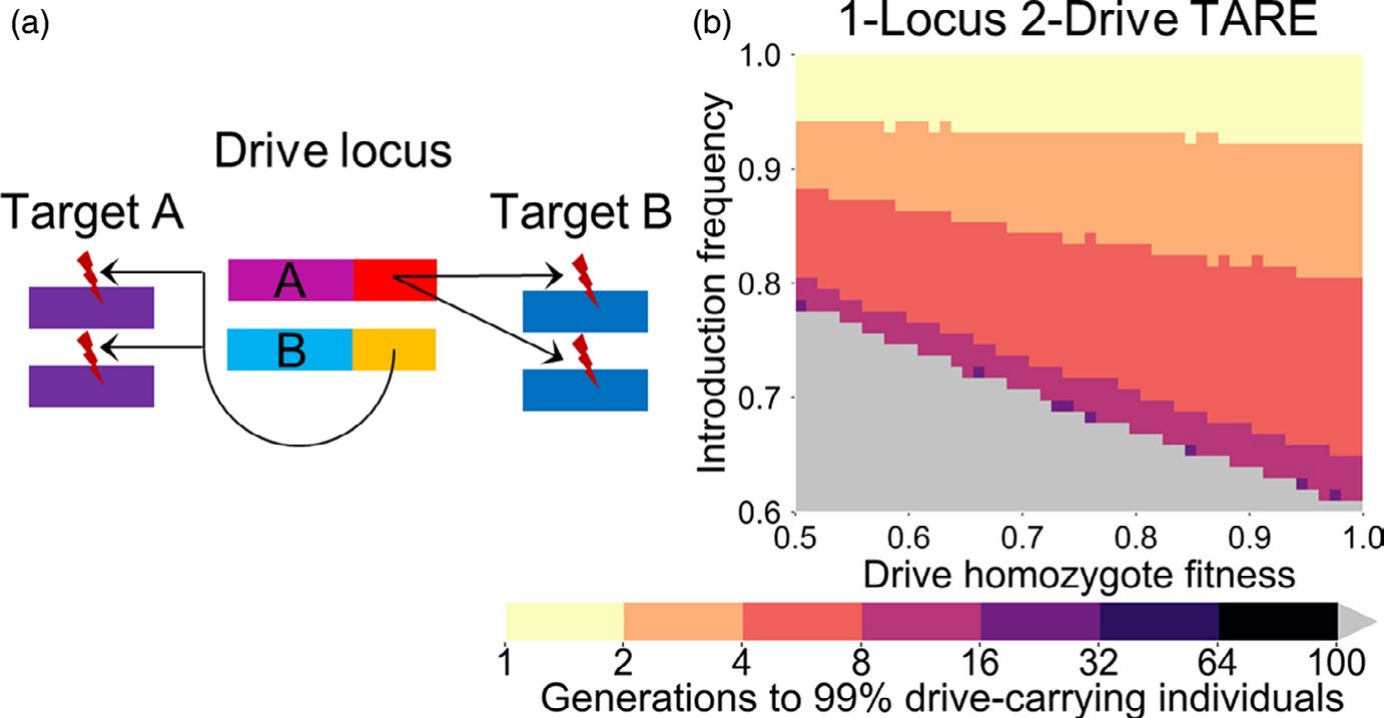
1‐locus 2‐drive TARE. (a) In the 1‐locus 2‐drive system, two TARE drive alleles (a and b) are situated at the same locus. Each targets a different essential but haplosufficient gene while providing rescue for the other drive allele's target. (b) The time at which a TARE‐based drive with a GE promoter is expected to reach 99% of individuals in the population in our simulation model with varying drive‐carrying individual introduction frequency and drive fitness. Released individuals have one copy of each drive allele at the drive site. Gray indicates that the drive was eliminated within 100 generations

We first propose a system that comprises two TARE drive alleles that occupy the same genetic locus (Figure 1a). A similar RNAi‐based system has an invasion threshold frequency of 67% (Champer, Zhao, et al., 2020; Davis et al., 2001; Dhole et al., 2018; Edgington & Alphey, 2018; Khamis et al., 2018). Each of the two drives targets an essential but haplosufficient gene. The first drive targets the gene which the second provides rescue for (via a recoded copy that cannot be targeted by the drive), and vice versa. One of these drive alleles could be in a same‐site configuration, or both could be in distant‐site configurations. We specifically model the latter system, along with a promoter that yields high Cas9 cutting activity in both the germline and in the early embryo from maternally deposited Cas9, which should be most efficient for standard TARE drives (Champer, Kim, et al., 2020). If the promoter also has somatic activity, then the dynamics of this system should be similar to the 1‐locus 2‐drive RNAi‐based system that has been modeled previously (Champer, Zhao, et al., 2020; Davis et al., 2001; Dhole et al., 2018; Edgington & Alphey, 2018; Khamis et al., 2018; this system has a slightly higher introduction threshold than the CRISPR 1‐locus 2‐drive TARE system).

The reciprocal targeting scheme of the drives, along with the fact that both drive alleles are situated at the same genomic locus, means that after all wild‐type alleles at the two target sites in a population have been disrupted, the only viable genotype is one in which both drive alleles are present (Table S1). Furthermore, all crosses involving females with a copy of each drive will result in some offspring not surviving (half in crosses with males with one copy of each drive, and all in crosses with wild‐type males). Thus, after fixation, this drive imposes a genetic load of 0.5 on the population even if the drive carries no additional fitness cost (see Methods). This will likely result in a modest suppressive effect if the drive alleles fixate, depending on the ecological characteristics of the organism in question. Because of these dynamics, this drive has the highest threshold of all the modification drives we analyze in this study. With a GE promoter, the drive requires an introduction above 61% to spread in the absence of additional fitness cost (63% for a G promoter). However, the drive can remove wild‐type alleles quickly when present at such high frequencies, resulting in the combined drive allele frequency rapidly reaching 100% if released above its threshold (Figure 1b).

#### 3.2.2 2‐locus drives

**Figure 2:**
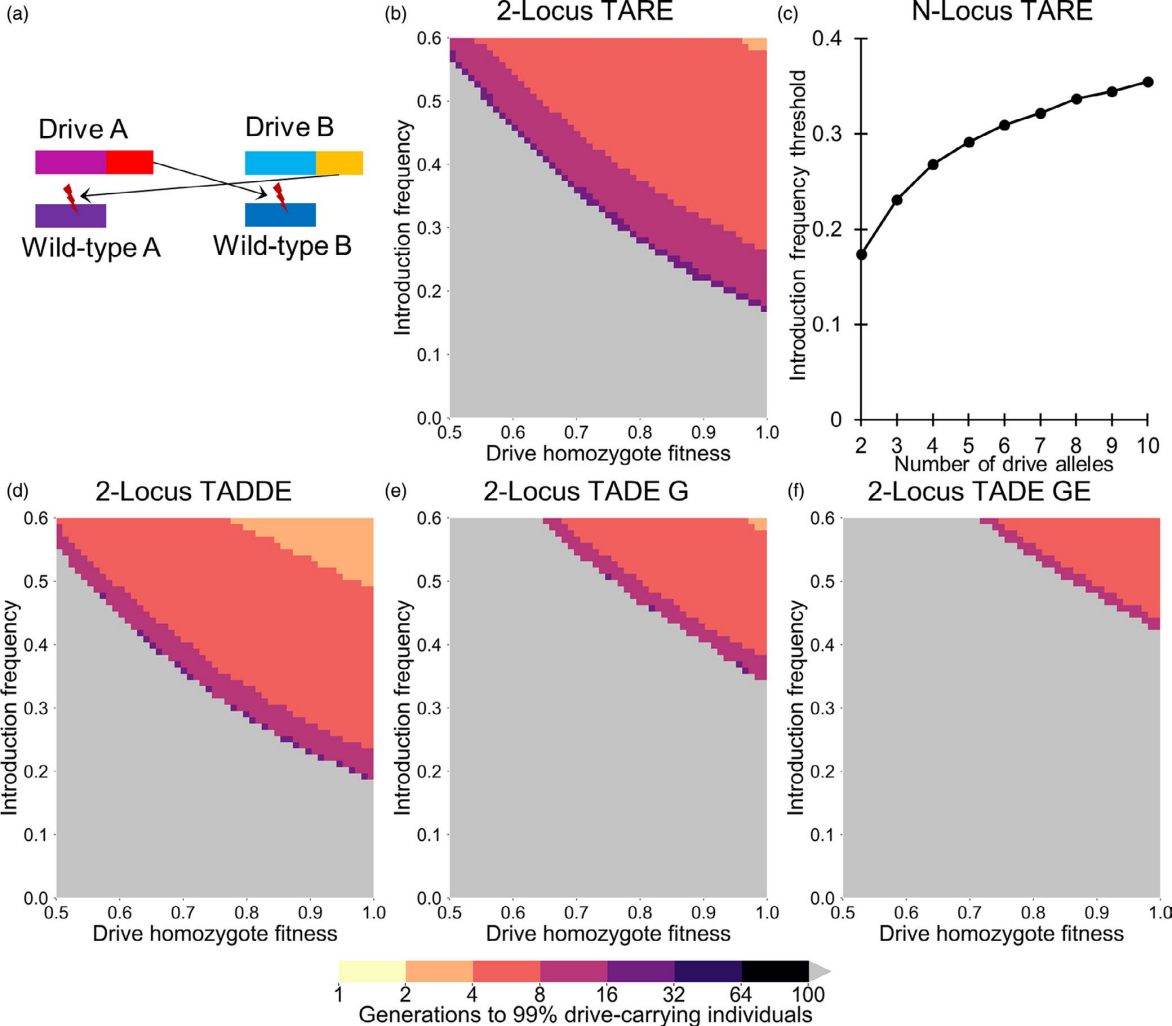
2‐locus drives. (a) In the 2‐locus drive systems, two drive alleles (both providing “same‐site” rescue in this example) each target an essential but haplosufficient gene while providing rescue for the other drive allele's target. (b) The time at which a 2‐locus drive with unlinked, same‐site TARE alleles with a GE promoter is expected to reach 99% of individuals in the population with varying introduction frequency and drive fitness. (c) The introduction frequency thresholds for TARE drives with additional loci, where each drive cyclically provides rescue for the target of the previous drive. (d) As in (b), but for TADDE alleles. (e) As in (b), but for TADE alleles with a germline (G) promoter. (f) As in (b), but for TADE alleles with a promoter causing cutting activity in the germline of both sexes and in embryos of drive‐carrying females (GE). Released individuals are homozygous for all drive alleles. Gray indicates that the drive was eliminated within 100 generations

We next propose a set of drives that comprise TARE, TADE, and TADDE alleles at two genetic loci, with each locus containing a different drive allele (thus allowing any or all the drives to be same‐site). Such a system based on RNAi elements has already been well‐studied, with an invasion threshold of 27% in the absence of fitness costs (Champer, Zhao, et al., 2020; Davis et al., 2001; Dhole et al., 2018; Edgington & Alphey, 2017, 2018; Huang et al., 2011; Khamis et al., 2018; Magori & Gould, 2006). As in the 1‐locus 2‐drive system, each drive allele targets the gene for which the other provides rescue (Figure 2a). However, because each drive allele has its own locus, there are relatively fewer possible parental combinations that result in the removal of drive alleles compared to wild‐type alleles, substantially decreasing invasion threshold frequencies compared to the 1‐locus drive arrangements. For example, if both drives are TARE type with GE promoters (Table S2), crosses between wild‐type males with heterozygous (at both loci) females result in 3/4 of offspring being nonviable, removing wild‐type and drive alleles at a 5:1 ratio. Crosses between two heterozygotes result in 7/16 of offspring being nonviable, removing wild‐type and drive alleles at a 5:2 ratio. Because of the relatively high rate of wild‐type allele removal, this drive has only a modest invasion threshold of 18% in the absence of fitness costs. The drive rapidly reaches all individuals (Figure 2b), but as in a single‐locus TARE drive, it takes longer for drive alleles to fixate, and the drive frequency will instead reach an equilibrium if drive alleles have a fitness cost (Champer, Kim, et al., 2020). The drive has similar performance with a somatic GES promoter but is somewhat slowed if a germline‐only promoter is used (though in the latter case, the invasion threshold is also slightly reduced to 17%).

A drive system can also be implemented with more than just two loci and types of drive alleles. In this case, each drive targets the gene that the next drive provides rescue for, with the last drive targeting the first. Figure 2c shows the invasion threshold frequencies of such systems based on TARE alleles. As the number of drives increases, the number of drive alleles removed by mutual disruption increases, thus increasing the threshold frequency.

In a 2‐locus TADDE drive, the doubled rescue element results in unchanged performance when using a promoter with or without embryo activity, or even a promoter with somatic expression in the offspring. This drive spreads slightly faster than the 2‐locus TARE drive since wild‐type alleles are removed more quickly (Figure 2d). Crosses between a drive/wild‐type heterozygote at both loci with a wild‐type individual result in 3/4 of offspring being nonviable, representing a removal of wild‐type and drive alleles at a ratio of 5:1 (Table S3). Crosses between two heterozygotes at both loci results in 7/16 of offspring being nonviable and represents a removal of wild‐type and drive alleles at a 5:2 ratio. These dynamics result in an invasion threshold of 19% in the absence of drive fitness cost, slightly higher than the TARE version.

A 2‐locus TADE system has substantially different performance with a G (Figure 2e) or GE (Figure 2f) promoter. With a G promoter, all crosses involving homozygotes have a regular number of offspring. However, with a GE promoter, crosses between any drive‐carrying female and a wild‐type male yield no offspring. A cross between a heterozygote female and a wild‐type male, when the drive utilizes a G promoter, results in 3/4 of offspring being nonviable, representing a removal of wild‐type and drive alleles at a 5:1 ratio (Table S4). A cross of heterozygotes at both loci results in only 1/16 of offspring being viable, representing a removal of wild‐type and drive alleles from the population at an 8:7 ratio. These drive dynamics result in an invasion threshold of 33% when using a G promoter and 43% when using a GE promoter.

#### 3.2.3 2‐locus TADE suppression

**Figure 3:**
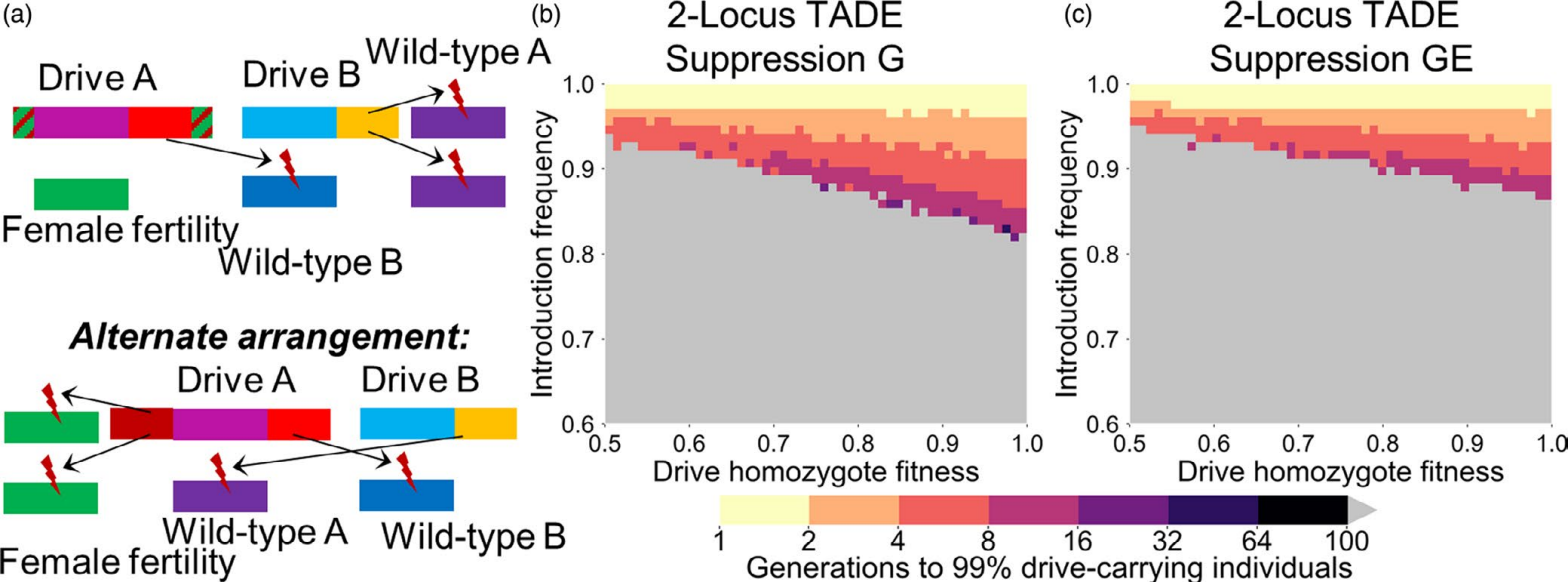
2‐locus TADE suppression drives. (a) A 2‐locus TADE drive will function as a suppression drive if one of its drive alleles is “distant‐site” and located in an essential but haplosufficient female fertility gene (or any single‐sex fertility or viability gene), disrupting the gene with its presence. Alternatively, the drive allele can simply target the fertility gene with additional gRNAs, allowing a “same‐site” arrangement. (b) The time at which a 2‐locus TADE suppression drive (with one allele placed in a female fertility gene and the other allele in a same‐site configuration, with all components genetically unlinked) with a germline promoter (G) is expected to reach 99% of individuals in the population with varying introduction frequency and drive fitness. (c) As in (b), but for TADE alleles with a promoter leading to cutting activity in the germline of both sexes and in embryos of drive‐carrying females (GE). Released individuals are heterozygous for all drive alleles. Gray indicates that the drive was eliminated within 100 generations

We next propose a 2‐locus TADE system wherein one of the drive alleles is placed in a female fertility gene (Figure 3a). This drive is potentially able to induce a high genetic load on a population, resulting in effective population suppression (Figure S2). In an alternative construction, a drive allele could instead target a female fertility gene with additional gRNAs (without providing rescue) and have similar suppressive performance. For suppression drives, we model heterozygote releases, since heterozygotes may be easier to generate and are fully fertile (the system technically has better performance if released males are homozygous and released females are heterozygous for the suppression element and homozygous for the second). As with the modification system, the drive system works best with a G promoter (Figure 3b) and has a higher invasion threshold and slower spread with a GE promoter (Figure 3c). In both cases, the invasion thresholds for the suppression variant are substantially higher than for the modification variant. Due to the many drive alleles that are in nonviable or sterile individuals, the drive has a relatively high invasion threshold of 83% with a G promoter and 88% with a GE promoter. The application of these systems would therefore require very large release sizes and only be practical in isolated areas where the drive would not be overwhelmed by the influx of wild‐type migrants.

#### 3.2.4 TADE underdominance systems

**Figure 4:**
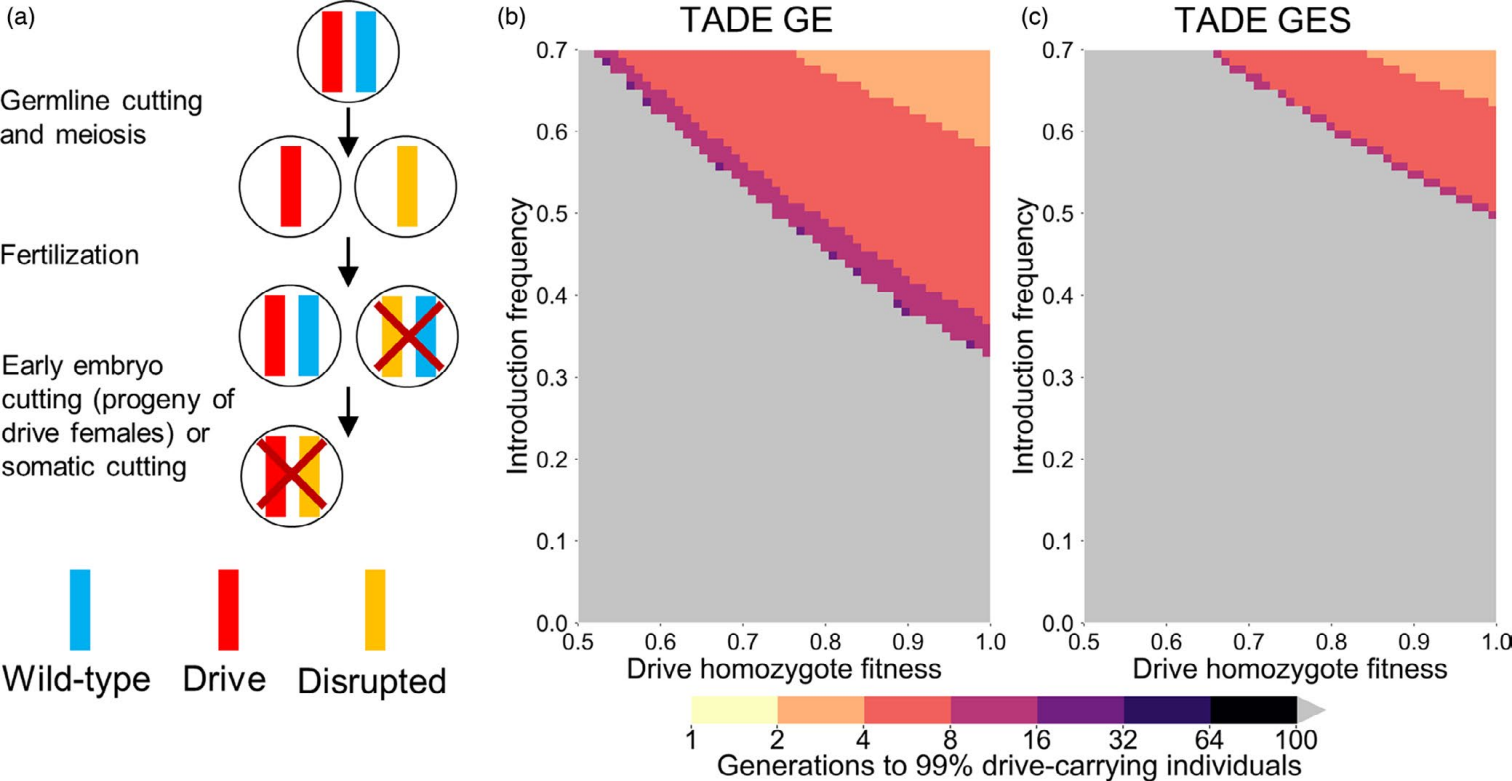
TADE underdominance drives. (a) A TADE drive acts as an underdominance drive if it utilizes a promoter that induces cutting activity in the germline and early embryo (GE) due to maternally deposited Cas9 and gRNA. The promoter can also have leaky somatic expression (GES), resulting in cutting of wild‐type alleles in somatic tissues by the drive. (b) The time at which a same‐site TADE drive with a GE promoter is expected to reach 99% of individuals in the population with varying introduction frequency and drive fitness. (c) As in (b), but for TADE alleles with a promoter that also drives somatic cutting (when this takes place in our model, such individuals are all nonviable). Released individuals are homozygous for the drive allele. Gray indicates that the drive was eliminated within 100 generations

We next propose a set of TADE underdominance systems. These drives are similar to the previously considered TADE drive (Champer, Kim, et al., 2020), but use a GE or GES promoter (Figure 4a). Such drives are expected to exhibit underdominance characteristics (unlike the standard TADE drive, which uses a G promoter) as well as a threshold. Here, we consider same‐site systems, but a similar system based on a distant‐site location with a GE promoter has been modeled previously and has highly similar characteristics (Oberhofer et al., 2019).

As implemented with a GE promoter, offspring from crosses between a drive homozygous male and wild‐type female are all viable, while crosses between a drive‐carrying female and a wild‐type male produce no offspring (Table S5). A male heterozygote crossed with a wild‐type female results in half the offspring being viable, removing only wild‐type alleles from the population. A cross between heterozygotes results in 3/4 of potential offspring being nonviable, producing only homozygotes and representing a removal of wild‐type and drive alleles at a 2:1 ratio. This drive has a moderate introduction threshold of 1/3 in the absence of allelic fitness costs, and above this threshold it spreads rapidly to fixation (Figure 4b).

When a TADE underdominance drive uses a GES promoter that also exhibits somatic activity, heterozygotes are nonviable, and crosses between drive and wild‐type individuals are assumed to never produce viable offspring. Therefore, in the absence of fitness costs, the drive has a 50% invasion threshold, but it still spreads rapidly when released above its threshold (Figure 4c). Such a system would have highly similar dynamics to single‐locus single‐allele RNAi‐based systems (Altrock et al., 2010, 2011; Láruson & Reed, 2016).

#### 3.2.5 TADE underdominance suppression

**Figure 5:**
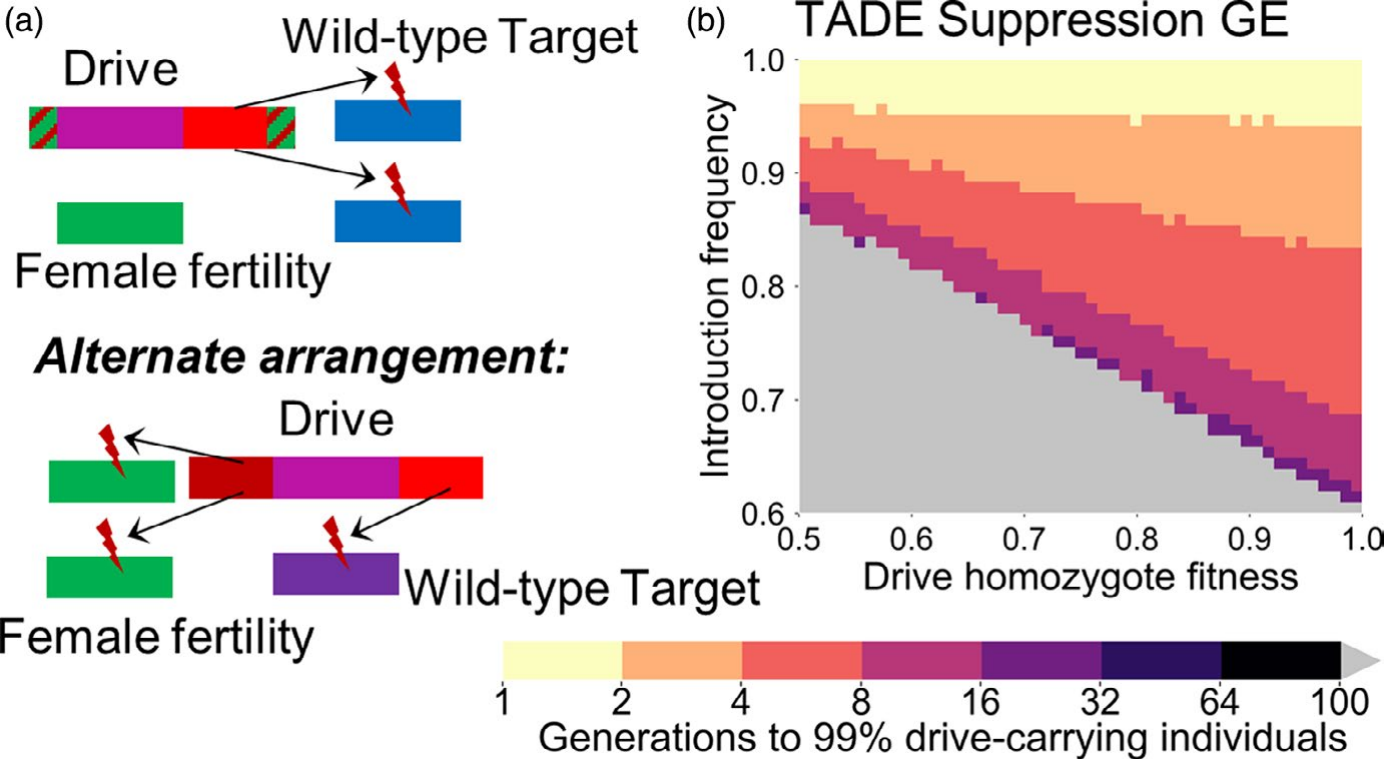
TADE underdominance suppression drive. (a) A TADE underdominance drive (with a GE promoter for activity in the germline and early embryo if the mother had a drive) will function as a suppression drive if one of its drive alleles is “distant‐site” and located in an essential but haplosufficient female fertility (or any single‐sex fertility or viability gene), disrupting the gene with its presence. Alternatively, the drive allele can simply target the fertility gene with additional gRNAs, allowing a “same‐site” arrangement. (b) The time at which a TADE underdominance suppression drive (placed in a female fertility gene) is expected to reach 99% of individuals in the population with varying introduction frequency and drive fitness. Released individuals are heterozygous for the drive allele. Gray indicates that the drive was eliminated within 100 generations

We next propose a TADE underdominance suppression drive. This drive can take the form of a distant‐site TADE underdominance drive with a GE promoter located inside a haplosufficient but essential female fertility gene (or other suitable location), disrupting the gene with its presence (Figure 5a). Alternatively, the drive can be same‐site with the haplolethal target and have additional gRNAs targeting the female fertility gene without rescue (Figure 5a). These two architectures were found to have similar performance. Because of the relatively many individuals that are nonviable or sterile from the effects of the drive, TADE underdominance suppression has a high invasion threshold frequency of 61% in the absence of drive fitness cost (Figure 5b).

#### 3.2.6 Additional TADE underdominance drive variants

It is possible that a particular promoter may have an intermediate level of cutting activity in the early embryo from maternally deposited Cas9 (Champer et al., 2017, 2018; Champer, Wen, et al., 2019; Hammond et al., 2018; Kyrou et al., 2018). A TADE modification or suppression drive configured with such an intermediate promoter has a lower threshold than one with a GE promoter, with the exact level being dependent on rate of embryo cutting (Figure S3A,B). Drives with these promoters spread rapidly, though less when the introduction frequency is close to their invasion threshold (Figure S4). While this potentially increases the range of invasion thresholds and confinement for a drive if the embryo cut rate can be adjusted, such an approach should be treated with caution. This is because for intermediate levels of embryo cutting, genetic background can significantly impact the cutting rate on an individual‐to‐individual basis (Champer et al., 2017; Champer, Wen, et al., 2019), and this could result in a change of the average rate over time as individuals with higher rates tend to be rendered nonviable more often.

In cases with no embryo cutting, a TADE suppression drive behaves similarly regardless of whether it is located in a male or female‐specific fertility gene. However, as the embryo cut rate increases, the invasion and migration thresholds for drives in a male fertility gene will increase more rapidly than the threshold for drives located in female infertility genes (Figure S3A,B). If Cas9 deposition is paternal, the patterns between male and female fertility genes are reversed. Such paternal deposition is observed far less often than maternal deposition, but it was confirmed in an X‐shredder in *Anopheles* based on I‐PpoI (Windbichler et al., 2008) and potentially has been seen at a lower level in *Anopheles* homing drives based on Cas9 (Beaghton et al., 2019; Kyrou et al., 2018)—though in the latter the results could potentially be explained by somatic expression of the nuclease. If either parent can deposit Cas9 into the embryo, then thresholds are further increased. All of these variants are still capable of placing a substantial genetic load though if either the germline or embryo cut rates are sufficiently high (Figure S3C). However, if a male fertility gene is used or if there is biparental embryo deposition, drives with sufficiently high embryo cut rates are not viable (Figure S3C).

Another variant is for the target gene to have an intermediate level of haploinsufficiency, thus placing it between the haplosufficient target of a TARE drive and the haplolethal target of a TADE drive. For a GE promoter, an increase in the degree of haploinsufficiency will steadily increase the invasion and migration thresholds from zero to the level of our TADE underdominance drives (Figure S5A,B). We also considered both modification and suppression drives in which both the target haploinsufficiency and the cut rate in the early embryo were allowed to vary (Figure S6). When haploinsufficiency is low, a GE promoter results in faster drive spread, and when haploinsufficiency is high, a G promoter is optimal for maximizing the rate of drive spread. The optimal level of embryo cutting transitions quickly from 100% to 0% at an intermediate level of haploinsufficiency (Figure S6A,B). This is usually around 0.2–0.5, with the exact level of the transition varying based on the specific type of drive (modification vs. suppression) and the initial release frequency. However, the rate of embryo cutting has little effect on the rate of spread of the drive in this intermediate region, as long as the initial release level is above the invasion threshold, which is dependent on both the embryo cut rate and the degree of target haploinsufficiency. Note that as the degree of haploinsufficiency decreases, a suppression drive would induce a lower genetic load on a population (Champer, Kim, et al., 2020), independent of the embryo cut rate.

#### 3.2.7 TAHRE modification and suppression drive

**Figure 6:**
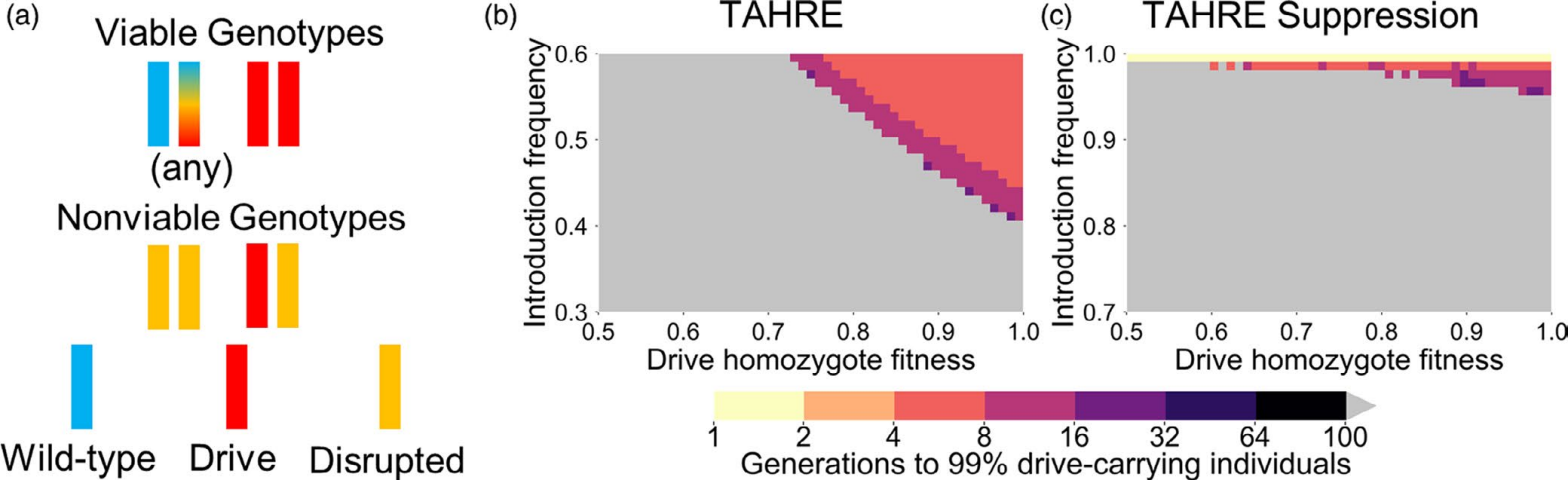
TAHRE drive. (a) A TAHRE drive uses a TARE target, but two drive copies are required to provide rescue, thus creating an underdominance system. (b) The time at which a same‐site TAHRE drive is expected to reach 99% of individuals in the population with varying introduction frequency and drive fitness. Released individuals are homozygous for the drive allele. (c) As in (b), but for a TAHRE suppression drive placed in a female fertility gene. Released individuals are heterozygous for the drive allele. Gray indicates that the drive was eliminated within 100 generations

We finally propose a TAHRE drive system. This drive targets an essential but haplosufficient gene and provides only half rescue (if no wild‐type copies of the target gene are present, two copies of the drive are required for viability). Thus, in contrast to a TARE drive, drive/disrupted target heterozygotes are nonviable (Figure 6a). This system is similar in concept to the previously proposed *Merea* drive based on RNAi (Marshall & Hay, 2012b). We consider a version of the drive with a GE promoter (Figure 6b).

Because the drive only provides half rescue, no viable offspring can result from a pairing between a drive‐carrying female and a wild‐type male (Table S6). Drive homozygote and heterozygote males produce a regular number of offspring with wild‐type females. Crosses between heterozygotes result in 3/4 of offspring being nonviable, representing removal of wild‐type and drive alleles at a 2:1 ratio. These characteristics result in the drive requiring a moderate invasion threshold frequency of 41% in the absence of drive fitness cost, which is the same as the *Merea* system (Marshall & Hay, 2012b; the invasion threshold is reduced to 35% if a G promoter is used).

When this drive utilizes a promoter that exhibits somatic CRISPR nuclease activity, all drive/wild‐type heterozygotes are nonviable, resulting in an invasion threshold frequency of 50% in the absence of drive fitness costs and identical characteristics to a TADE drive with a similar promoter (Figure 4c).

We also propose that a TAHRE drive can be converted to a suppression system in the same manner as described for the TADE drives (Figure 6c). In this case, the drive has a very high invasion threshold frequency of 96% in the absence of fitness costs (the system is nonfunctional with a G or GES promoter). While this threshold is high, such a system is still a gene drive and would theoretically have substantially more suppression power than methods involving sterile insect technique (Harris et al., 2012) or releases of *Wolbachia*‐carrying males (Alphey et al., 2013).

#### 3.2.8 Target genes with incomplete lethality when disrupted

Thus far, we have considered target genes in which lack of at least one (TARE) or two (TADE) functional wild‐type target or drive allele copies must be present to avoid lethality. However, for some possible target genes, instead of a lethal effect, individuals may survive but suffer negative fitness effects, which we term “incomplete lethality”. Here, we consider drives with such targets. Instead of nonviability, individuals with insufficient rescue are always fully viable, but they have their fitness (male mating success and female fecundity, as defined in the methods) multiplied by a fixed value between zero (corresponding to full sterility for females and lack of mating ability for males) and one (where disruption of the target gene has no effect). If the drive has a TADE target, the full fitness reduction is only suffered by drives lacking any functional wild‐type target or drive alleles, and individuals with one such allele have their fitness multiplied by the square root of this fixed value.

In general, drives with incomplete lethality targets have lower invasion thresholds (Figure S7). However, when the fitness of individuals with incomplete rescue is sufficiently high, the construct is no longer able to function as a gene drive (Figure S7). Additionally, higher levels of fitness for individuals with incomplete rescue also slows the spread of the drive (Figure S8). This is because the rate of removal of disrupted target alleles is reduced, thus slowing the rate at which the drive increases in relative frequency. Suppression type drives are sufficiently slowed that, even when released above their invasion threshold, they will reach an equilibrium frequency instead of going to fixation. This reduces the potential genetic load that a suppression drive induces in a population (Figure S9).

### 3.3 Confinement of underdominance TA systems

#### 3.3.1 Modification drives

**Figure 7:**
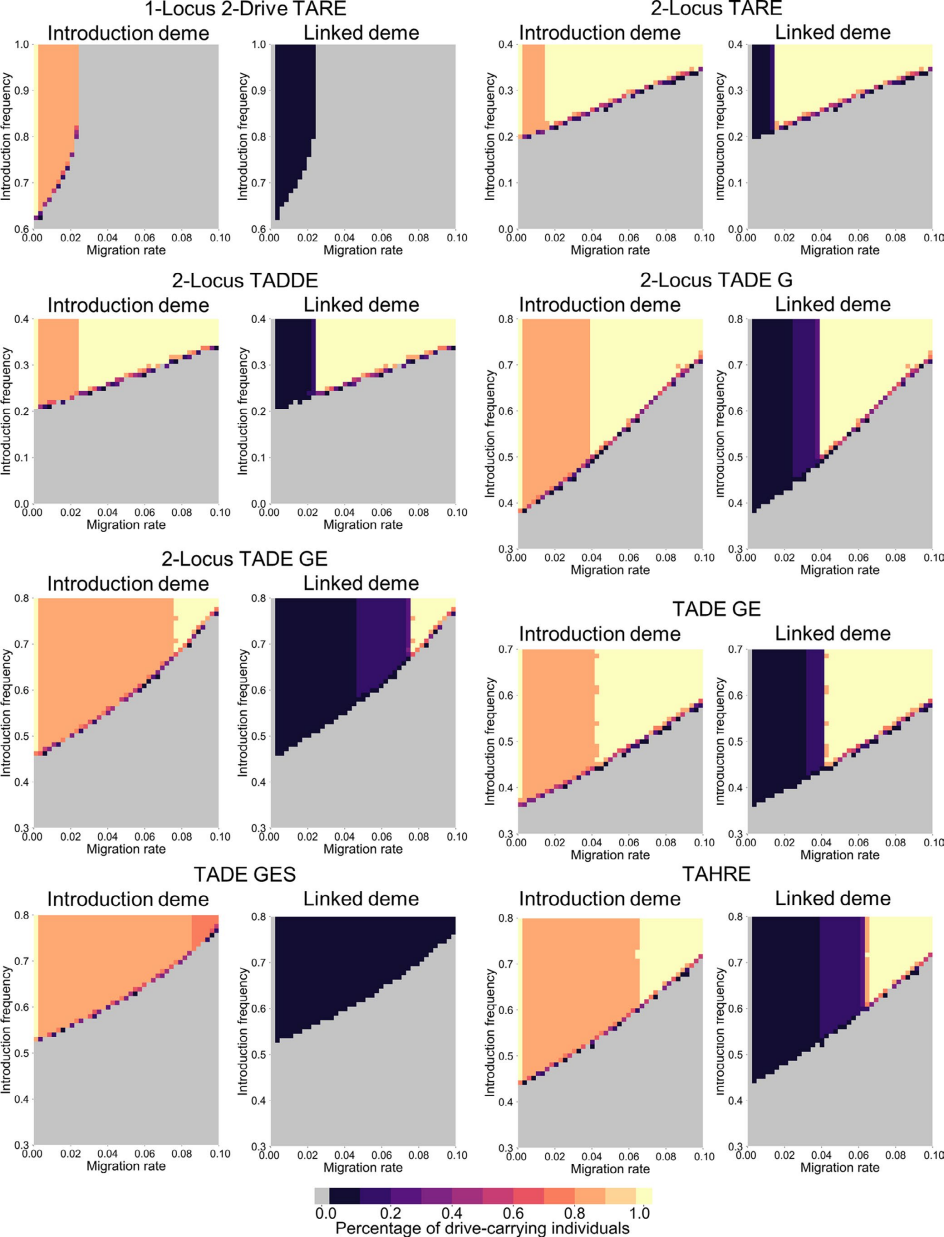
Modification drives in a 2‐deme model. Each drive is released at a variable introduction frequency in the first deme, which is linked to the second deme by a variable per‐generation migration rate. Released individuals are homozygous for the drive allele. The frequency of drive‐carrying individuals in each deme is shown as an average of the frequencies between the 100th and 150th generations after the drive is released

To further assess potential confinement of our CRISPR underdominance TA gene drives, we modeled a system in which a drive was introduced into one of two separate demes with equally sized populations linked by symmetric migration (Figure 7). This could potentially represent a drive release into a “target” population along with an adjacent “nontarget” population, where it is desirable to prevent the drive from reaching high frequency. In these scenarios, possible outcomes included elimination of the drive, substantial spread of the drive in only the introduction deme, and substantial spread of the drive in both demes.

Each of the drives exhibited an introduction frequency, as in the single‐deme simulations, above which the drive would increase in frequency and below which the drive would be eliminated. Higher migration rates increased the introduction threshold needed for the drive to successfully spread in the first deme due to influx of wild‐type individuals from the second deme. At lower levels of migration, all of the drives, if they were released above the introduction threshold, remained largely confined to the first deme. In this case, an equilibrium was formed in which only a low frequency of drive alleles was present in the second deme (stabilizing at a value determined by the rate of migration and rate of allele removal of the particular construct). In the introduction deme, an influx of wild‐type individuals from the second deme prevented any of the drives from reaching all individuals, but the equilibrium frequency was generally high. Releasing the drive at higher frequencies yielded no change in the simulations, with the drive frequency in the target deme quickly reaching the same equilibrium value.

However, at higher levels of migration, most of the drives were able to spread into the second deme, eventually reaching all individuals in both demes. The TADE drive with a GES promoter and the 1‐locus 2‐drive TARE system, however, could not substantially spread into the second deme at any migration rate, since their usual introduction thresholds are at or over 50% (note that this would not necessarily hold if the population sizes in both demes were not equal). In general, drives with lower introduction thresholds in single‐deme scenarios were able to spread to the second deme with lower migration levels. While we focused our analysis on type of drive, migration rates, and introduction frequencies, note that the outcome of a drive release in a two‐deme scenario would also be influenced by the promotor used, as well as the fitness impact of the drive, as in the single‐deme scenarios.

#### 3.3.2 Suppression drives

**Figure 8:**
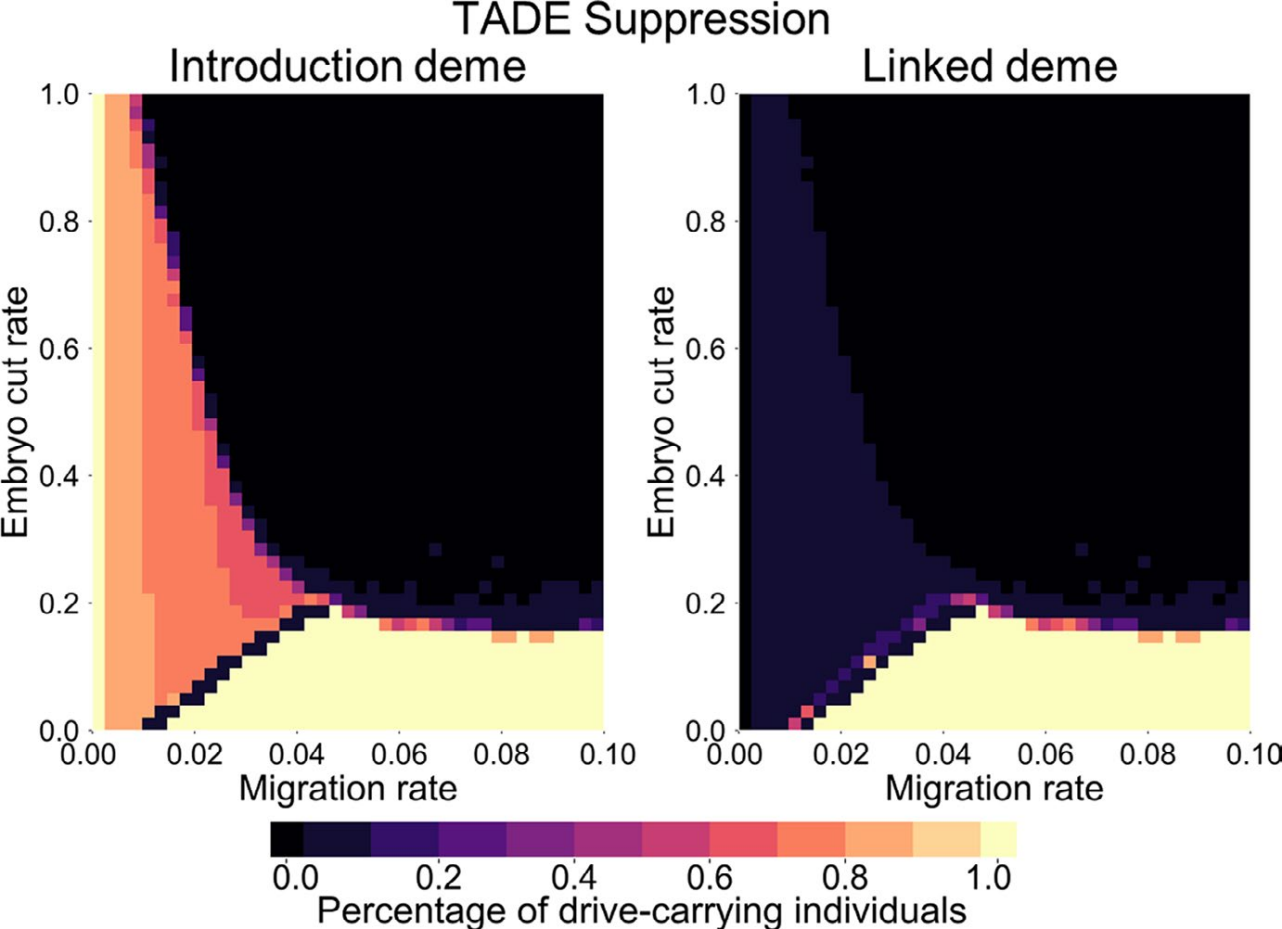
TADE suppression drive in a 2‐deme model. A TADE suppression drive (placed in a female fertility gene) with a variable embryo cut rate is released at 70% frequency in the first deme (a rate that is high enough to allow the drive to successfully suppress the population of the first deme in the absence of migration between the two demes regardless of the embryo cut rate), which is linked to the second deme by a variable per‐generation migration rate. Released individuals are heterozygous for the drive allele. The average frequency of drive‐carrying individuals in each deme is shown between 100 and 150 generations after the drive is released. Yellow color (100% frequency) indicates complete population eradication

Suppression drives are potentially easier to confine, since the population decreases as the drive increases in frequency, resulting in a corresponding reduction to the number of emigrants. However, for the same reason, these drives are also more sensitive to incoming migration of wild‐type individuals. For all migration rates analyzed at or above 0.0025 (the lowest nonzero rate assessed), both forms of 2‐locus TADE suppression and TAHRE suppression drives were quickly eliminated from the introduction population in our 2‐deme scenarios. Single‐locus TADE suppression with a GE promoter was able to establish with a high release frequency and low level of migration (Figure 8). However, it was unable to eradicate the population in the face of continual migration from the second linked deme. We also analyzed other promoter forms by allowing the embryo cut rate to vary (Figure 8). When the cut rate is low, such as with a G promoter, the drive is able to establish, and when the embryo cut rate is below 20%, sufficiently high migration allows the drive to spread and eradicate the population in both demes. Higher release sizes would tend to slightly increase the parameter range in which the drive can establish and achieve an equilibrium (though the equilibrium itself would not be substantially affected), while lower release sizes would increase the parameter range in which the drive fails to establish. When population eradication in both demes is not possible, the drive is never able to significantly spread in the linked deme. In the introduction deme, an intermediate effective genetic load after drive establishment (Figure S10) results in partial suppression of the population (i.e., partial population suppression takes place and is confined to the introduction deme).

With bidirectional migration between a target and nontarget population, confined eradication may not be a realistic possibility since recolonization of the empty deme would quickly take place. We thus assessed additional scenarios in which migration only takes place from the target population to the nontarget population. Here, population suppression or eradication in the first deme is unaffected by the second deme. For most drives presented in this study, any drive alleles in the linked deme are quickly eliminated. However, a TADE suppression drive with a G promoter is able to spread to the second deme and eradicate the population if the migration is high enough (Figure S11). Of note, higher initial introduction frequency reduces the number of drive individuals that migrate into the second deme before the population in the first deme is eradicated. Thus, higher initial drive introduction can effectively prevent establishment of the drive and eradication of the population in the second deme (Figure S11).

## 4 DISCUSSION

In this study, we have presented several new CRISPR‐based underdominance TA gene drive designs that could allow for the development of localized population modification or suppression drives (Table 1). Such systems can utilize a broad class of target genes and promoters, suggesting that their construction may be feasible in many target organisms. Indeed, two examples of CRISPR‐based TA systems have already been demonstrated experimentally (Champer, Lee, et al., 2020; Oberhofer et al., 2019), and a similar TA underdominance system would likely only require a rearrangement of existing components (combining these two or using similar alleles with swapped gRNAs to form a 2‐locus TARE drive). Systems using haplolethal targets may be somewhat more difficult to engineer due to the sensitivity of organisms to these gene's expression levels. They are likely feasible, though, since the targeting of such a gene was recently demonstrated experimentally for a homing drive (Champer, Yang, et al., 2020). On the other hand, it remains unclear how TAHRE rescue elements would be constructed.

**Table 1:** Comparison of CRISPR TA drive types

Drive type	Threshold^a^	Suppression threshold^b^	Promoter	Engineering difficulty
TARE ^(^ ^e^; ^f^; ^g^ ^)^	Low	N/A	Any	Proven (Champer, Lee, et al., 2020; Oberhofer et al., 2019)
TADE (^e^)	Low	Low	G	Medium
TADDE ^(^ ^e^ ^)^	Low	N/A	Any	Medium?
TADS ^(^ ^e^ ^)^	Zero	Zero	Any	High?
1‐locus 2‐drive TARE	High	N/A	Any	Low
2‐locus TARE	Medium	N/A	Any	Low
2‐locus TADE	Medium	Very High	G,GE	Medium
2‐locus TADDE	Medium	N/A	Any	Medium?
TADE Underdominance	Medium	High^c^	GE, GES^d^	Low?
TAHRE	Medium	Very High	G, GE	High?

Note

Blue shading indicates drives with high thresholds (likely allowing “safe” use in a wide variety of scenarios), drives that can flexibly use many different types of promoters, and drives that are anticipated or demonstrated as easier to engineer. Red represents drives without introduction thresholds and that use restricted promoters and are difficult to engineer. Yellow represents intermediate levels of these attributes.

Abbreviations: G, germline‐only promoter; GE, promoter with germline and early embryo cutting (in the progeny of drive‐carrying females); GES, promoter that induces a high rate of somatic cleavage.

^a^ Thresholds (for both modification and suppression) assume a small drive fitness cost and provide an indirect measure for the degree of confinement.

^b^ These are for suppression variants of the drive. N/A indicates that a strong suppression form of the drive is not possible.

^c^ A TADE Underdominance suppression system could have a “medium” threshold if it had intermediate early embryo cutting.

^d^ A strong suppression drive cannot use a GES promoter.

^e^ Champer, Kim, et al., 2020

^f^ Champer, Lee, et al., 2020

^g^ Oberhofer et al., 2019

From among these possible configurations, TARE‐based systems may likely be the preferred candidate in many situations. Compared to previously proposed RNAi‐based gene drive systems, CRISPR‐based TA systems would avoid the difficulty of working with RNAi while usually having slightly lower introduction threshold frequencies for similar configurations (Champer, Kim, et al., 2020; Champer, Zhao, et al., 2020). Such drives are also presumably the easiest to engineer of CRISPR TA systems [they are based on elements that have already been successfully engineered (Champer, Lee, et al., 2020; Oberhofer et al., 2019)], and allow for a variety of thresholds depending on the arrangement of drive components. TADE drives are more difficult to engineer due to the required manipulation of a haplolethal target gene (and thus, possible loss of a large fraction of transformed insects after embryo microinjection, unless more complicated strategies are used), but they could still be the preferred drive system if there is a need to more rapidly generate drive homozygous individuals in the target population (possibly due to a payload gene requiring two copies for full effectiveness). TADE drives may also be the preferred system for suppression‐based approaches. In these cases, a single‐locus system would usually be the easiest to engineer, and would still have a stringent level of confinement (at least 50% given a high embryo cutting rate). Care should be taken if a lower level of confinement for a suppression drive is desired, as an intermediate level of embryo cutting in the laboratory may prove to be highly variable in wild, genetically diverse populations (Champer et al., 2017; Hammond et al., 2015).

Compared to homing drives, TA underdominance systems allow for local confinement, have lower rates of resistance allele formation by avoiding reliance on the error‐prone process of homology‐directed repair (Champer, Kim, et al., 2020), and should have a reduced rate of mutational inactivation of potential payload genes, since these are copied only by replication rather than the more error‐prone homology‐directed repair process. Note, however, that care must still be taken in the construction of such systems to avoid the formation of resistance alleles by undesired homology‐directed repair of rescue elements (and not other drive elements). Procedures to mitigate the formation of this type of resistance have already been successfully demonstrated in both same‐site (Champer, Lee, et al., 2020) and distant‐site (Oberhofer et al., 2019) configurations of TA elements. An additional advantage of most TA systems over homing drives is that they often do not require a germline‐specific promoter due to their ability to tolerate maternal deposition and subsequent activity of Cas9 in the embryo. Some TA systems can even tolerate ubiquitous Cas9 somatic activity.

**Figure 9:**
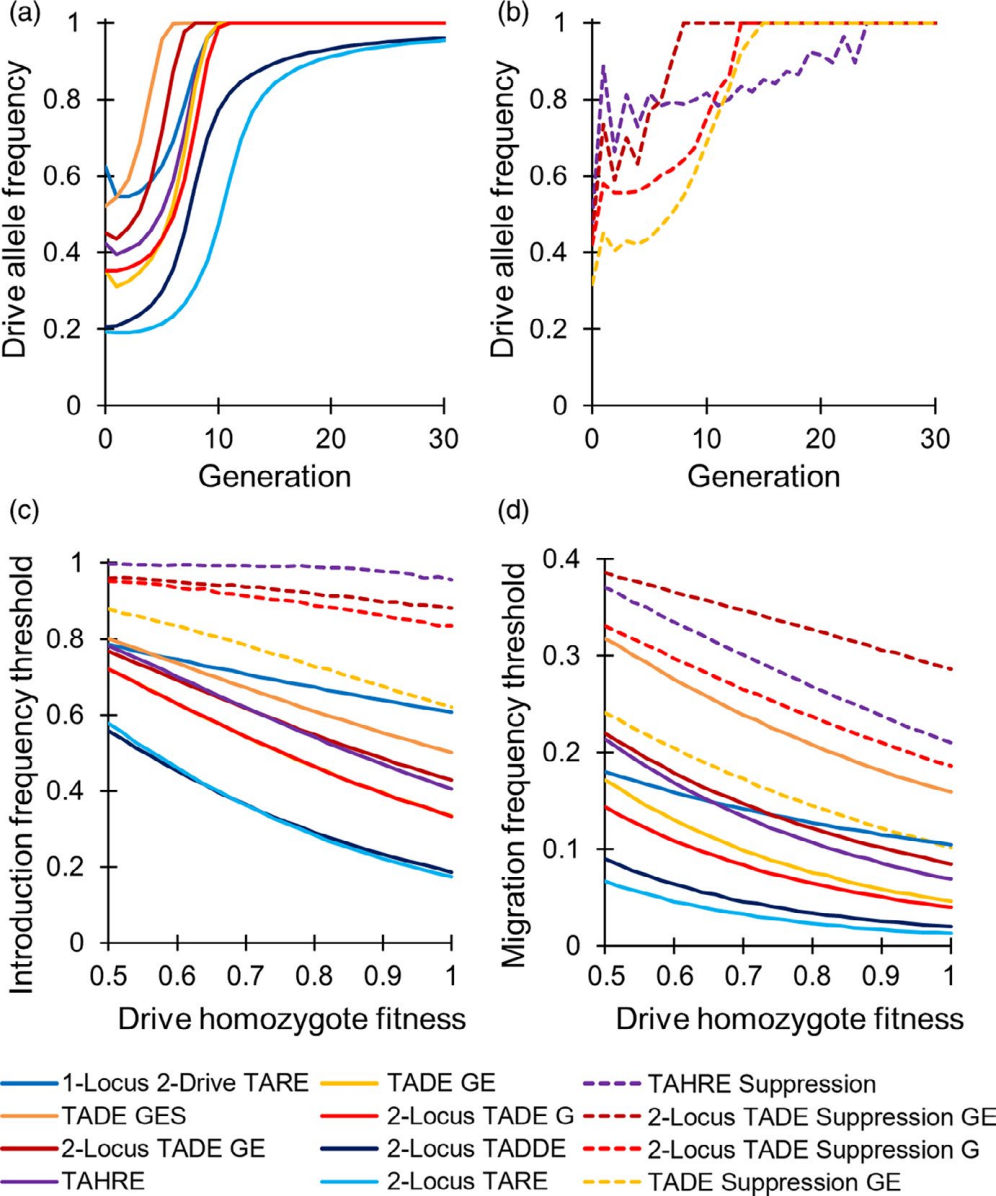
Dynamics of TA underdominance drives. (a) Example allele frequency trajectories for modification drives introduced at 2% above their introduction frequency thresholds in our population model. (b) Example allele frequency trajectories for suppression drives introduced at 2% above their invasion threshold frequency. (c) Invasion frequency thresholds (the frequency of introduced drive‐carrying individuals, as a fraction of the initial population, above which the drive will increase in frequency and below which the drive will be eliminated) and (d) Migration frequency thresholds (the per‐generation rate of migration of drive‐carrying individuals as a percentage of the initial population above which the drive will increase to a high frequency instead of remaining at a low equilibrium frequency). Note that the TADE GE and 2‐locus TADE G drives have the same thresholds. In modification systems, released individuals were homozygous for the drive. In suppression systems, individuals were heterozygous for the drive. G, germline‐only promoter; GE, promoter with germline and early embryo cutting (in the progeny of drive‐carrying females); GES, promoter that induces a high rate of somatic cleavage

The systems presented here are all “local” drives with relatively high invasion threshold frequencies even in the absence of drive fitness costs, spanning a wide range of thresholds (Figure 9). However, confinement in realistic environments is a more complex issue and cannot be completely addressed by the simple 2‐deme models presented herein. Though introduction thresholds can provide a means for comparing the potential confinement of different drive systems, the absolute confinement will depend on more complex ecological parameters. For example, modeling in continuous space has shown that drives with invasion threshold frequencies at or above 50% in panmictic populations can often fail to persist in well‐connected populations unless the drive is released over a wide area, while drives with invasion thresholds below 50% can be invasive in many scenarios (Barton, 1979; Barton & Turelli, 2011; Champer, Zhao, et al., 2020). Thus, the potential for “local” drives with a variety of invasion threshold frequencies [together with previously considered “regional” drives (Champer, Kim, et al., 2020; Champer, Lee, et al., 2020; Oberhofer et al., 2019)] should provide increased flexibility in the development of an appropriate drive for a given application, though additional high‐fidelity models will be needed to determine which drives have acceptable levels of confinement.

Though we have modeled a wide variety of drives in this study, additional combinations exist that might be suitable in some situations or easier to construct. For example, mutually targeting 2‐locus systems can be made up of any combination of individual drive type elements (TARE, TADE, etc). Suppression drives can utilize additional gRNAs targeting the female fertility gene instead of disrupting the gene by presence of the drive. This could allow a same‐site suppression drive to be more easily constructed, with the attendant advantages in rescue element efficiency (Champer, Lee, et al., 2020). Each of the proposed modification drive systems could be combined with a tethered homing drive (Dhole et al., 2019) to provide confinement based on the TA underdominance system, along with the power of a homing drive for strong suppression or to spread costly payloads efficiently. A TARE drive and a tethered homing system may also prove easier to engineer than TA drives with haplolethal targets.

We note that our study focused on a wide variety of different drive types, using a simplified ecological model for ease of comparison between drives. However, it should be noted that the ecological situation can have a substantial effect on the outcome of simulations. This is particularly true for suppression drives, where the strength of density dependence, distribution of the population, type and stage of density related competition, and other factors such as lifecycle and environmental characteristics can substantially influence the outcome of a gene drive release (Dhole et al., 2020). In particular, we assumed that fitness costs would reduce fecundity and mating success, though effects on egg‐to‐adult viability would undoubtedly be present as well, particularly since competition in many relevant species, including mosquitoes, often takes place at the larval stage.

Overall, our modeling analysis suggests that CRISPR‐based underdominance TA systems could be used for both population modification and suppression with a high number of possible variants with different invasion threshold frequencies. Their construction requires elements that have already been demonstrated (Champer, Lee, et al., 2020; Oberhofer et al., 2019), making the varieties presented here, together with “regional” TA systems (Champer, Kim, et al., 2020), promising candidates for the development of flexible strategies for confined gene drive. Future experimental and computational studies should further characterize underdominance TA drives and assess their implementation in potential target species.

## CONFLICT OF INTEREST

The authors declare that they have no conflict of interest.

## Supporting information

Table S1‐6

Supplementary Material

## Data Availability

The SLiM program and parameter files allowing the reader to reproduce all simulations presented here are available on GitHub (https://github.com/MesserLab/TA‐Underdominance‐Drives).
