# Supplementary material for: Design and analysis of CRISPR‐based underdominance toxin‐antidote gene drives
Source: Evol Appl. 2020 Dec 21;14(4):1052–69. doi: 10.1111/eva.13180 (PMC8061266; doi:10.1111/eva.13180)
Supplement: Supplementary file 2 — Supplementary Material [file EVA-14-1052-s002.pdf]

Design and analysis of CRISPR-based underdominance toxin-antidote gene drives

SUPPLEMENTAL INFORMATION

Parameter/variable list (value or range, if applicable)

germline cut rate (0-1)
embryo cut rate (0-1)
somatic activity (yes/no)
drive locus gene type (none, essential but haplosufficient female fertility, essential but haplosufficient male fertility)
rescue efficiency (full/half)
target gene type (haplosufficient but essential, haploinsufficient/haplolethal)
incomplete lethality level (0-1)
haploinsufficiency (0-1)
drive architecture (number of alleles, sites, etc.)
population carrying capacity (100,000)
presence of a second deme (yes/no)
migration frequency (0-0.4, either into single deme or between demes)
introduction size (0-1)
low-density growth rate (10)
number of female mating attempts (10)
number of bionomial trials for offspring generation (50)
drive fitness (0.5-1)

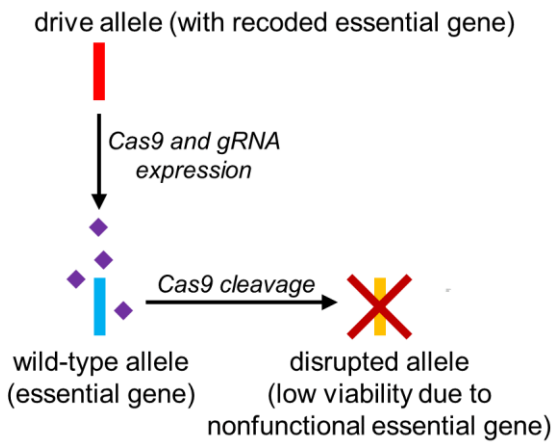

Figure S1. Mechanism of CRISPR toxin-antidote drives. The drive allele expresses Cas9 and gRNA, which cut the wild-type target allele (an essential gene), converting it into a disrupted allele. Disrupted alleles experience non-viability (with the details depending on the type of target allele) and are removed from the population, increasing the relative frequency of the drive allele.

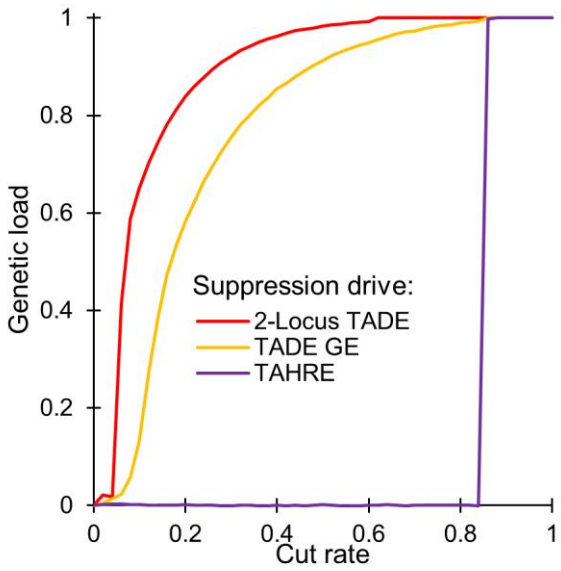

Figure S2. Genetic load of suppression drives. The genetic load imposed on a population as a function of the cut rate (in both the embryo and the germline) in the suppression drives we considered. We define genetic load as the fractional reduction in the population size of the next generation (caused by the drive at final equilibrium) compared to the expected next generation population size had the population during the present generation been composed entirely of wild-type individuals.

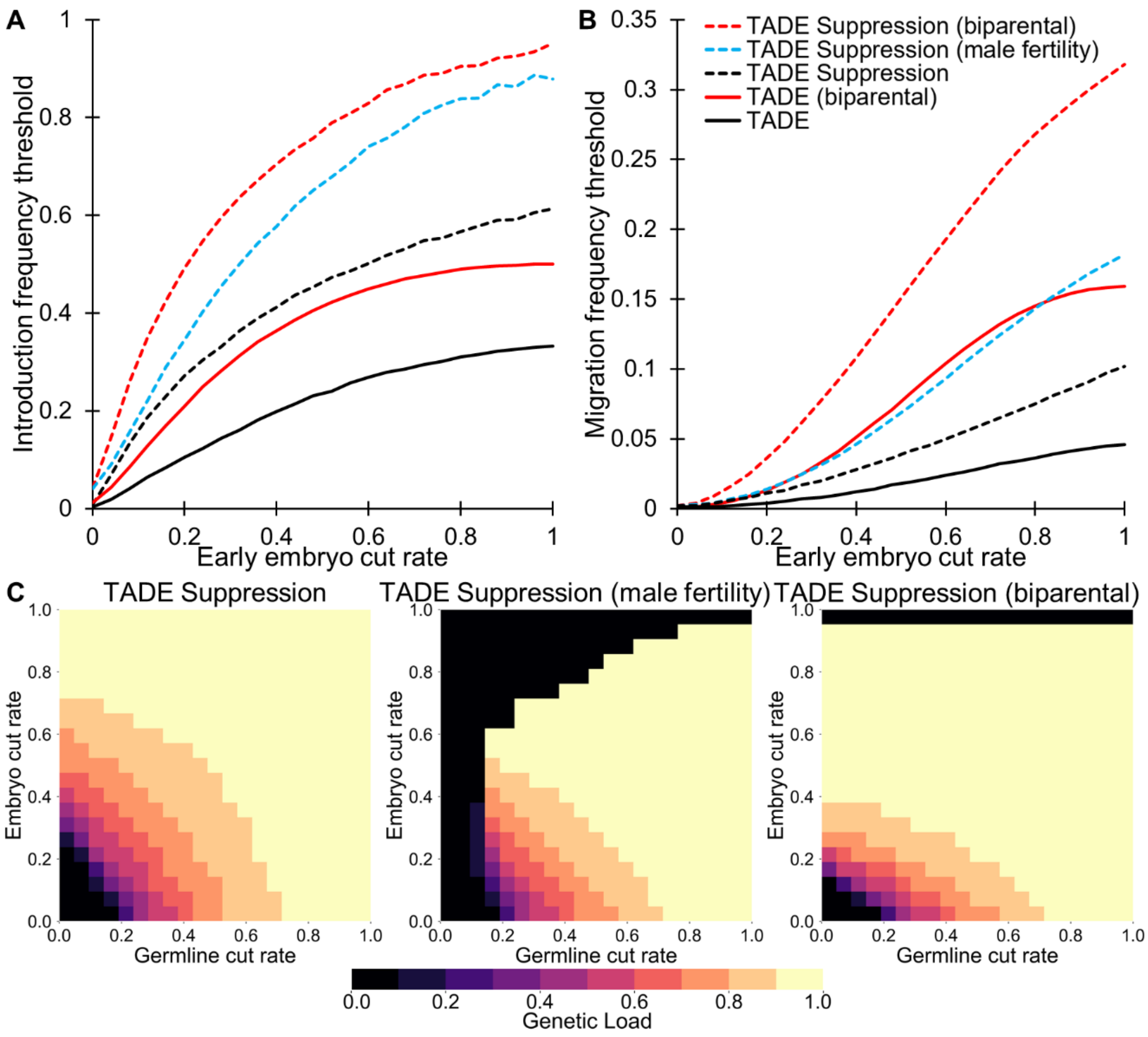

Figure S3. Effect of germline and embryo cut rates on TADE drive thresholds and genetic load. (**A**) Invasion or (**B**) migration threshold frequency in TADE drives (with a 100% germline cut rate) as a function of the early embryo cut rate. Released individuals are homozygous for the TADE drive and heterozygous for the TADE suppression drive. (**C**) The genetic load imposed on a population by TADE suppression drives as a function of the germline and embryo cut rates. The standard TADE suppression drive is placed in a female fertility gene and has only maternal (not paternal) Cas9 deposition into the embryo. "Male fertility" refers to drive designs placed inside a male fertility gene instead of a female fertility gene (a drive in a female fertility gene with only paternal and not maternal Cas9 deposition would have identical dynamics). "Biparental" refers to a drive placed in either a male or female (but not both) fertility gene with Cas9 deposition into the embryo by both male and female parents.

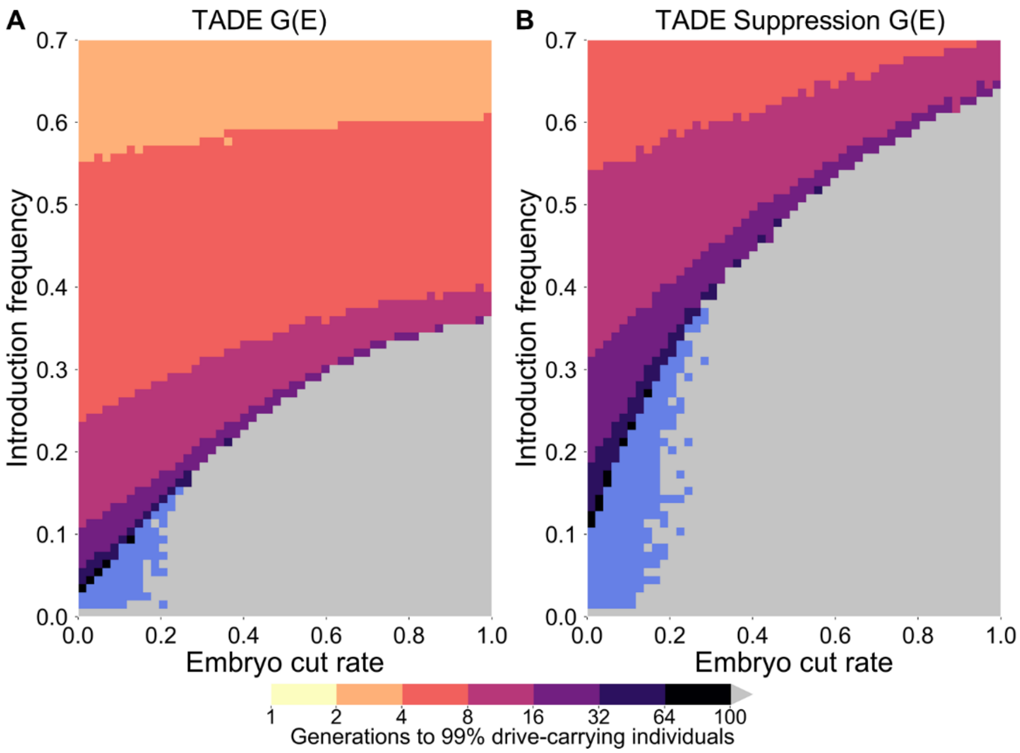

Figure S4. TADE drive performance with variable embryo cut rate. (**A**) The time at which a TADE drive is expected to reach 99% of individuals in the population with varying introduction frequency and embryo cut rate (in the progeny of drive-carrying females). Released individuals are homozygous for the drive allele. (**B**) As in (A), but for a TADE suppression drive (placed in a female fertility gene). Released individuals are heterozygous for the drive allele. Grey indicates that the drive was eliminated within 100 generations. The tan colored areas represent regions where the drive neither reaches 99% of individuals nor is eliminated within 100 generations (this occurs due to the low fitness cost of these drives, which results in drive alleles only being removed when together with a disrupted allele; this becomes more common as the embryo cut rate increases).

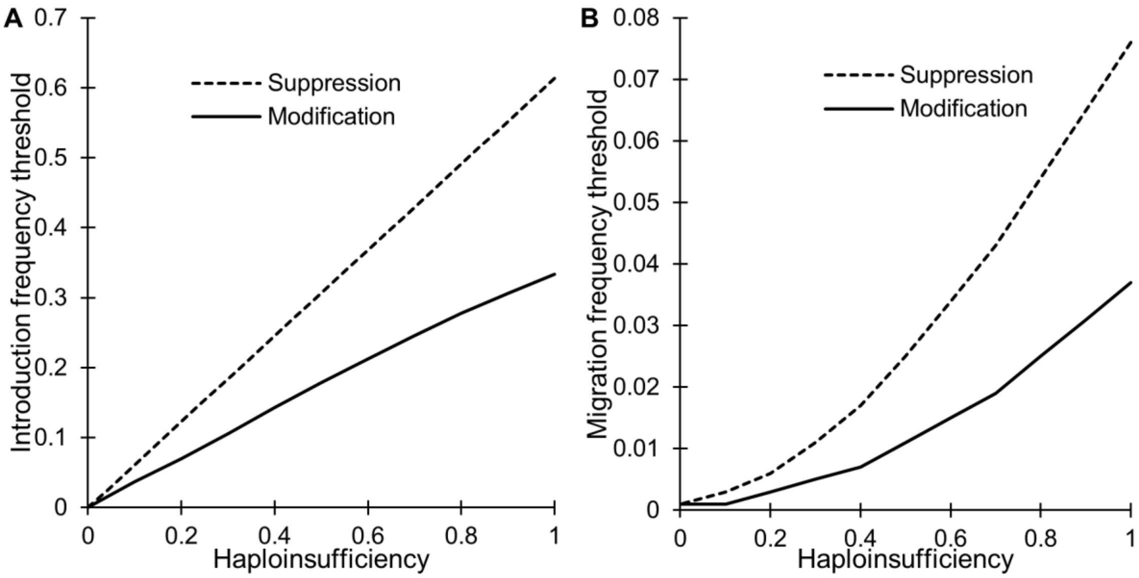

Figure S5. Effect of haploinsufficiency on drive thresholds. (**A**) Invasion (single-release) and (**B**) migration (release each generation) threshold frequency in single-locus TA drives as a function of the degree of target haploinsufficiency. Released individuals are homozygous for the drive allele for the modification drive and heterozygous for the suppression drive.

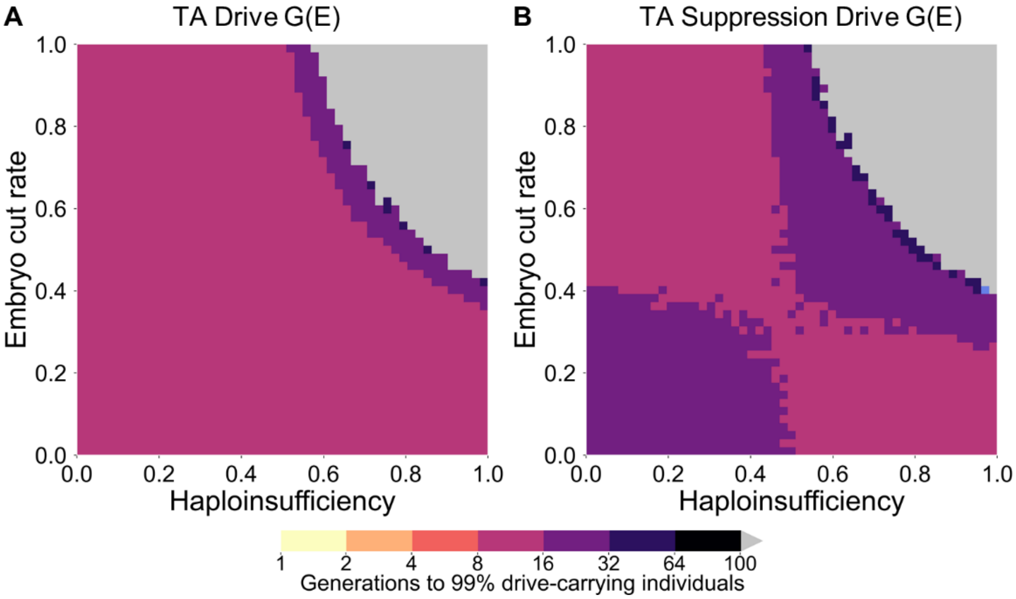

Figure S6. TA drive performance with variable haploinsufficiency and embryo cut rate. (**A**) The time at which a TA drive is expected to reach 99% of individuals in the population with varying haploinsufficiency and embryo cut rate (in the progeny of drive-carrying females). Homozygous individuals are released at 20% initial frequency. (**B**) As in (A), but for a suppression drive (placed in a female fertility gene) and with a release of heterozygous individuals at 40% initial frequency. Grey indicates that the drive was eliminated within 100 generations.

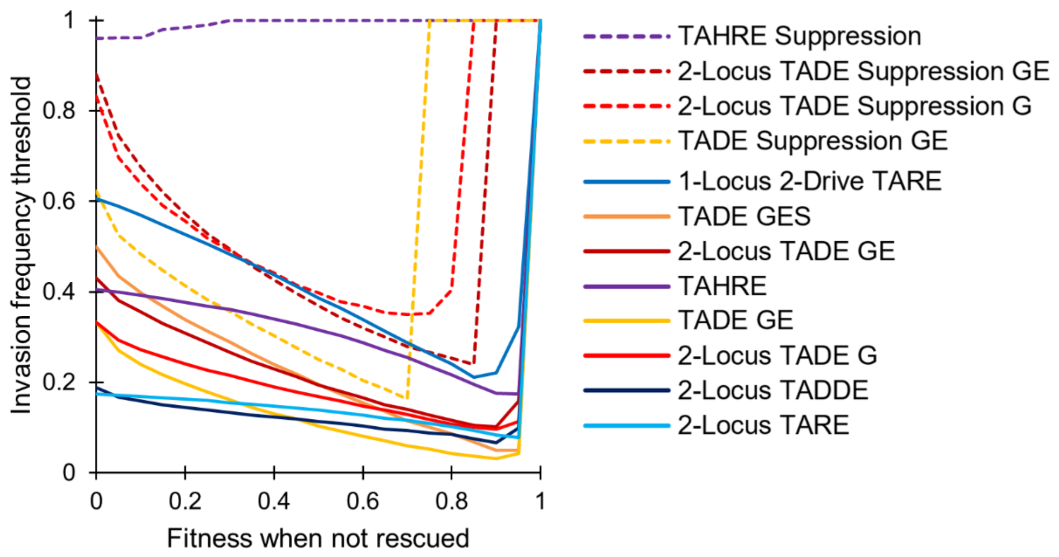

Figure S7. Invasion thresholds of TA underdominance drives with incomplete lethality. Invasion threshold frequencies as a function of the fitness of individuals where rescue is incomplete. In modification systems, released individuals were homozygous for the drive. In suppression systems, individuals were heterozygous for the drive. G = germline only promoter. GE = promoter with germline and early embryo cutting (in the progeny of drive-carrying females). GES = promoter that induces a high rate of somatic cleavage. A threshold of "1" indicates that the system is unable to function as a gene drive.

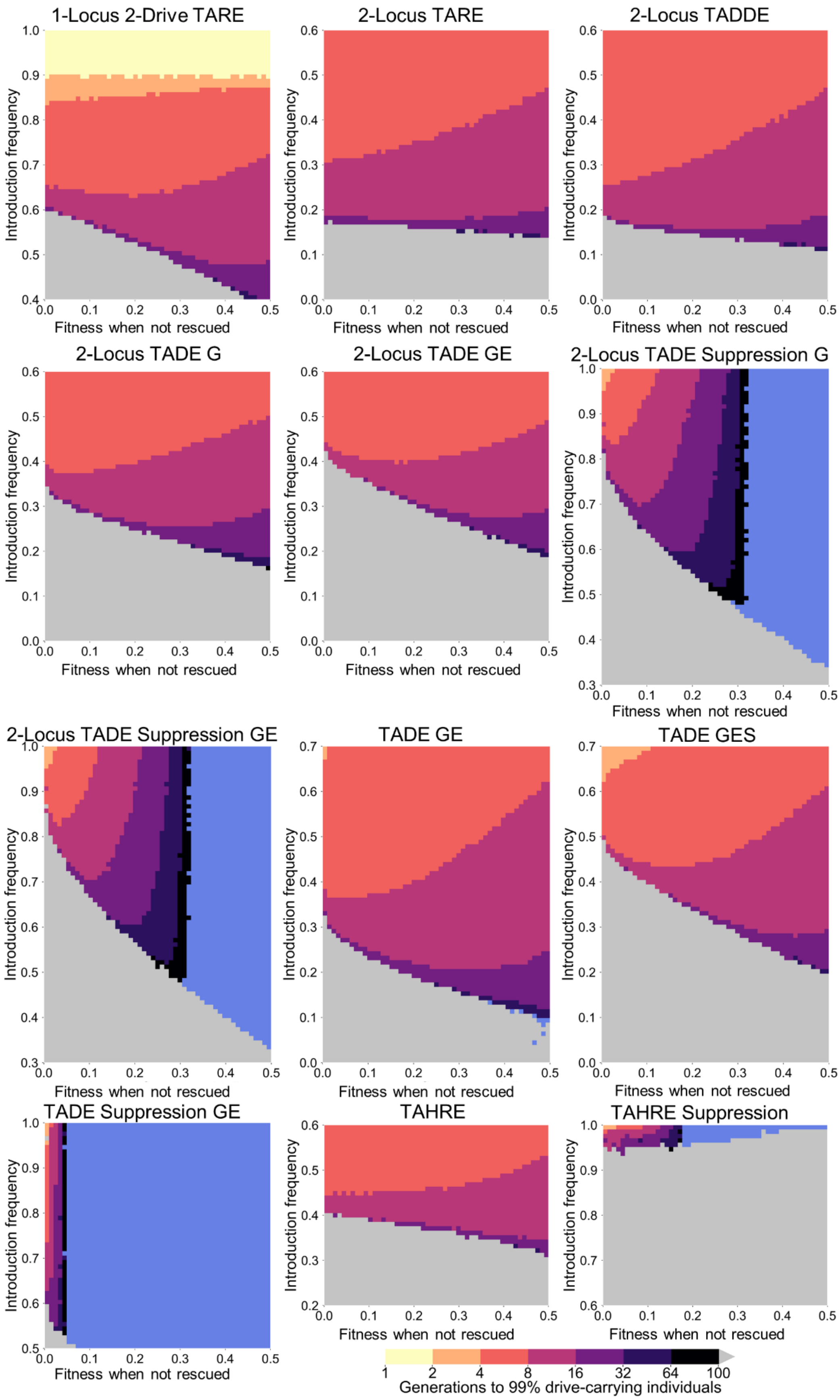
1-Locus 2-Drive TARE
2-Locus TARE
2-Locus TADDE
2-Locus TADE G
2-Locus TADE GE
2-Locus TADE Suppression G
2-Locus TADE Suppression GE
TADE GE
TADE GES
TADE Suppression GE
TAHRE
TAHRE Suppression
Introduction frequency
Fitness when not rescued
1
2
4
8
16
32
64
100
Generations to 99% drive-carrying individuals

Figure S8. Drive performance with incomplete lethality targets. Heatmaps show the time at which each drive is expected to reach 99% of individuals in the population with varying introduction frequency and embryo cut rate (in the progeny of drive-carrying females). Released individuals are homozygous for the drive allele for modification drives and heterozygous for suppression drives. Grey indicates that the drive was eliminated within 100 generations. The tan colored areas represent regions where the drive does neither reaches 99% of individuals nor is eliminated within 100 generations (usually representing a suppression drive that is able to spread and reach a high equilibrium frequency, but that cannot induce a sufficient genetic load to eradicate the population, see Figure S9).

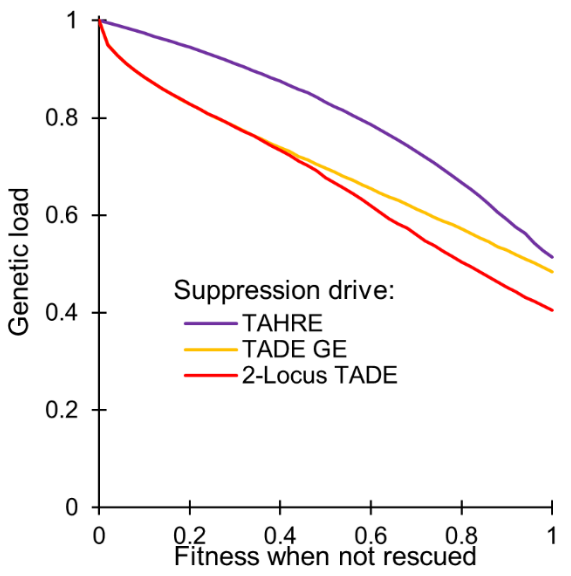

Figure S9. Genetic load of suppression drives with incomplete lethality. The genetic load imposed on a population as a function of the fitness of individuals where rescue is incomplete in the suppression drives we considered. We define genetic load as the fractional reduction in the population size of the next generation (caused by the drive at final equilibrium) compared to the expected next generation population size had the population during the present generation been composed entirely of wild-type individuals. Note that the 2-Locus TADE drive has the same genetic load for variants with a G or a GE promoter.

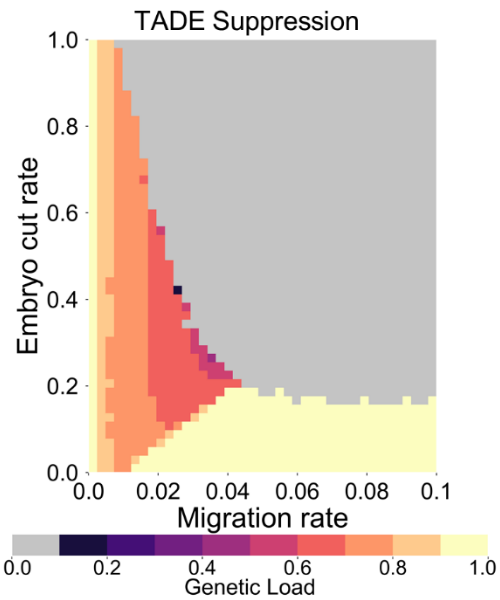

Figure S10. Genetic load in the introduction deme for TADE suppression drive in a 2-deme population. A TADE suppression drive (placed in a female fertility gene) with a variable embryo cut rate is released at 70% introduction frequency in the first deme, which is linked to the second deme by a variable per-generation migration rate. Released individuals are heterozygous for the drive allele. The genetic load of the drive in the first deme is shown as an average of that found between 100 and 150 generations after the drive is released (genetic load in the second deme is negligible for most of the parameter space, and equal to the first deme in the yellow area). Note that the genetic load is reduced due to continual migration from the second deme. The actual level of population reduction will depend on species- and ecology-specific factors.

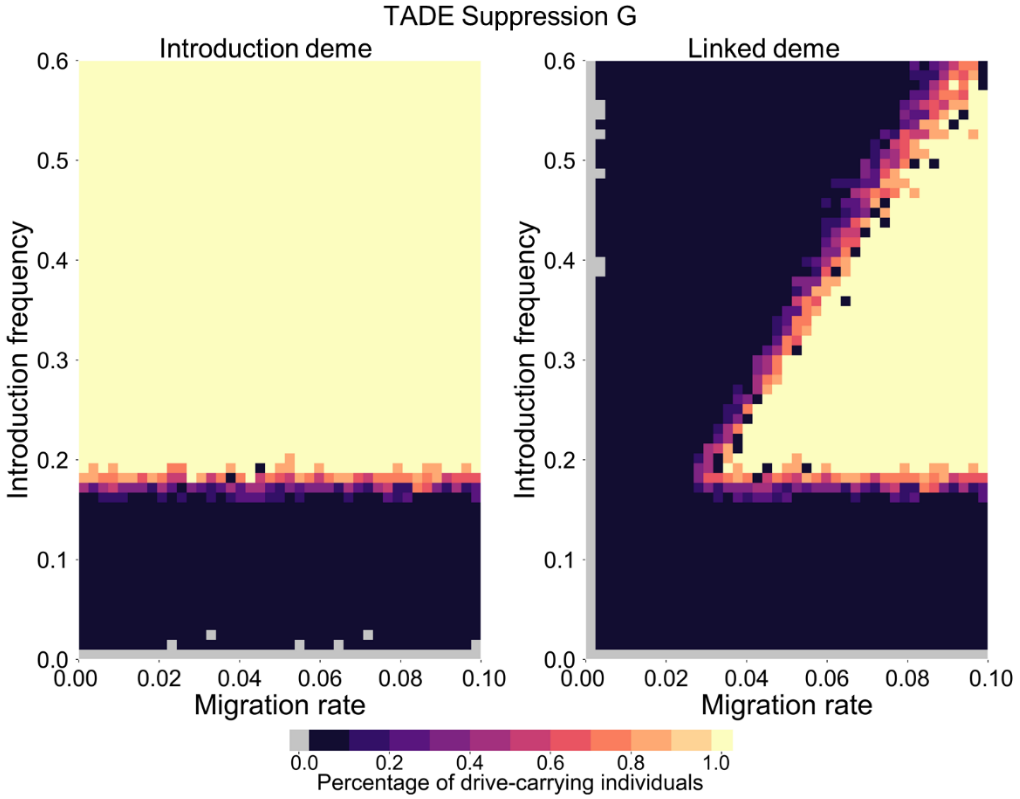

Figure S11. TADE suppression G drive in a 2-deme model with unidirectional migration. A TADE suppression drive (placed in a female fertility gene) with a G promoter is released at a variable introduction frequency in the first deme, which is linked to the second deme by a variable one-way, per-generation, migration rate, wherein individuals migrate from the target deme to the non-target deme, but not vice-versa. Released individuals are heterozygous for the drive allele. The frequency of drive-carrying individuals in each deme is shown as an average of the frequencies between the 100th and 150th generations after the drive is released. Yellow color (100% frequency) indicates complete population eradication.
